# Age-Related Alterations in Peripheral Immune Landscape with Magnified Impact on Post-Stroke Brain

**DOI:** 10.34133/research.0287

**Published:** 2023-12-11

**Authors:** Jianan Lu, Huaming Li, Guoqiang Zhang, Fan Yang, Xiaotao Zhang, An Ping, Zhouhan Xu, Yichen Gu, Rui Wang, Dan Ying, Jianjian Liu, Jianmin Zhang, Ligen Shi

**Affiliations:** ^1^ Department of Neurosurgery, Second Affiliated Hospital, School of Medicine, Zhejiang University, Hangzhou, Zhejiang, China.; ^2^ Clinical Research Center for Neurological Diseases of Zhejiang Province, Hangzhou, China.; ^3^ Brain Research Institute, Zhejiang University, Hangzhou, Zhejiang, China.; ^4^ Collaborative Innovation Center for Brain Science, Zhejiang University, Hangzhou, Zhejiang, China.

## Abstract

Immunosenescence refers to the multifaceted and profound alterations in the immune system brought about by aging, exerting complex influences on the pathophysiological processes of diseases that manifest upon it. Using a combination of single-cell RNA sequencing, cytometry by time of flight, and various immunological assays, we investigated the characteristics of immunosenescence in the peripheral blood of aged mice and its impact on the cerebral immune environment after ischemic stroke. Our results revealed some features of immunosenescence. We observed an increase in neutrophil counts, concurrent with accelerated neutrophil aging, characterized by altered expression of aging-associated markers like CD62L and consequential changes in neutrophil-mediated immune functions. Monocytes/macrophages in aged mice exhibited enhanced antigen-presentation capabilities. T cell profiles shifted from naive to effector or memory states, with a specific rise in T helper 1 cells and T helper 17 cells subpopulations and increased regulatory T cell activation in CD4 T cells. Furthermore, regulatory CD8 T cells marked by Klra decreased with aging, while a subpopulation of exhausted-like CD8 T cells expanded, retaining potent immunostimulatory and proinflammatory functions. Critically, these inherent disparities not only persisted but were further amplified within the ischemic hemispheres following stroke. In summary, our comprehensive insights into the key attributes of peripheral immunosenescence provide a vital theoretical foundation for understanding not only ischemic strokes but also other age-associated diseases.

## Introduction

The peripheral immune system plays a crucial and indispensable role in shaping the development and various functions of the central nervous system (CNS). It also contributes to the pathogenesis of CNS diseases, from the initial stages of acute injury through the subsequent recovery process [1,2]. Strategies involving the regulation of the peripheral immune system for the intervention in CNS diseases hold significant research value [3]. Aging inevitably impacts nearly every component of the immune system, which in turn influences the genesis and progression of CNS diseases [4–6]. However, the specific changes that aging induces in the immune system—a phenomenon known as immunosenescence—and their subsequent effects on the physiological functions and pathological processes within the CNS remain largely unexplored.

Immunosenescence is inherently complex and variable, with different immune cells displaying diverse responses to aging [4,7]. For instance, aging markedly alters the functionality and quantity of both neutrophils and natural killer (NK) cells. It reduces the microbicidal activity of neutrophils and diminishes the cytotoxic capabilities of NK cells, which, in turn, exacerbates infection and tissue damage [7–9]. Furthermore, the aging process shifts the inflammatory response by altering the composition of T cells and reducing the ratio of naive to memory T cells [10]. Despite these insights, research has largely been focused on macro-level changes in specific immune cell types, with scant attention to the nuanced alterations in various subpopulations.

Ischemic stroke is a cerebrovascular disease primarily affecting the elderly population [11,12]. Previous studies have demonstrated that in the context of aging, resident brain immune cells—microglia—exacerbate functional impairments following ischemic stroke [11,13]. Similarly, the aging process also has a significant impact on peripheral immune cells, affecting the prognosis of ischemic stroke. Specifically, aging increases the presence of procoagulant neutrophils, which obstruct cerebral microcirculation in ischemic regions and worsen outcomes. Aging also amplifies the infiltration of CD8 T cells, thereby potentiating the inflammatory response [14–16]. However, comprehensive studies investigating aging-induced changes in peripheral immune cells and their subsequent impact on stroke recovery are conspicuously absent.

In this study, we explored the intricacies of immunosenescence in the peripheral blood of aged mice, focusing on accelerated neutrophil aging, enhanced antigen-presenting functions in monocytes and macrophages, and shifts in T cell-mediated immunity. Notably, these age-associated changes are not confined to the circulatory system but are also manifest when these immune cells infiltrate cerebral ischemic regions following a stroke event.

## Results

### Immunosenescence hallmarks and the impact of aging on peripheral immune cell infiltration and functionality in post-stroke mice

**Fig. 1.**
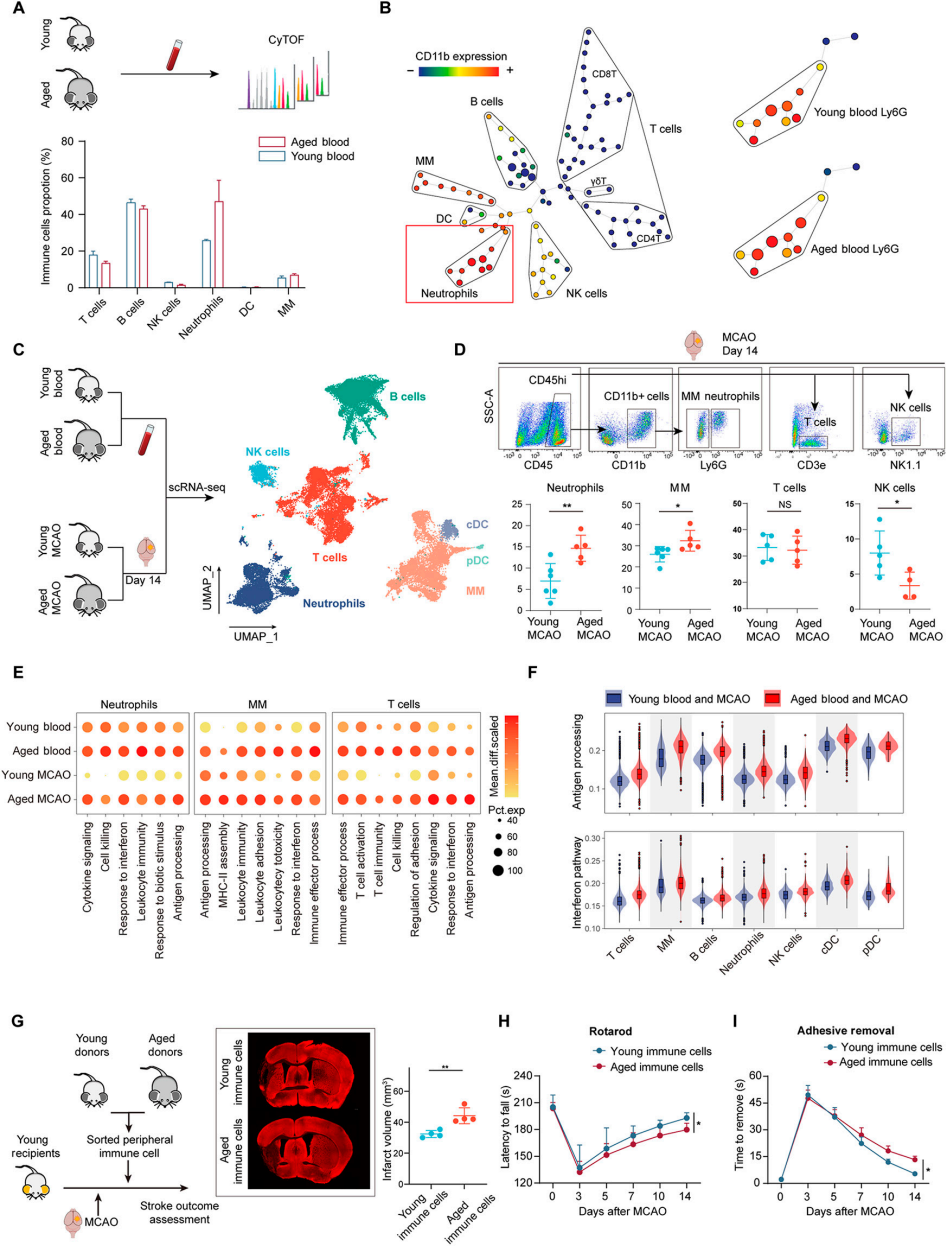
Characteristics of peripheral blood immune aging in mice and its impact on the immune microenvironment of the ischemic brain. (A) Peripheral blood immune cells from aged (*n* = 4) and young (*n* = 3) mice were isolated and subjected to CyTOF analysis (top). Proportion of various immune cell subsets within peripheral immune cells for each group (bottom). (B) Representative SPADE results of mass cytometry data with cluster size indicating cell proportion and color denoting CD11b expression levels (left); in the SPADE results, representation of neutrophil cell proportions in aged and young mice is displayed, identified by Ly6G as a marker (right). (C) Peripheral blood immune cells from aged and young mice, as well as CD45hi immune cells from both groups at 14 d post-MCAO, were sorted for scRNA-seq (left); UMAP projection plot showing cell clusters of immune cells in the periphery and ischemic brain of aged and young mice (right). (D) Flow cytometry analysis of the infiltrated immune cell populations in the ischemic brain of aged (*n* = 4 to 5) and young mice (*n* = 5 to 6) at 14 d post-MCAO. *P < 0.05, **P < 0.01, NS means not significant, Student *t* test. (E) Up-regulated functional pathways in neutrophils, MM, and T cells in the aged group compared to the young group. (F) Antigen-presenting functions and interferon pathways were significantly up-regulated in various immune cell populations in the periphery of aged mice compared to young mice. *P* ≤ 0.0001 in all immune cell clusters, Bonferroni-corrected Wilcoxon rank sum test. (G) Schematic of the experimental procedure in which recipient mice received peripheral immune cells from either aged or young donor mice; right panel: mice that received immune cells from aged donors showed larger infarct volumes 14 d post-MCAO compared to mice that received immune cells from young donors. *n* = 4 per group. **P < 0.01, Student *t* test. (H and I) Rotarod (H) and adhesive removal tests (I) suggest that mice received aged immune cells (*n* = 7 for Rotarod; *n* = 8 for adhesive removal) exhibited worse long-term sensory and motor function recovery than those received young immune cells (*n* = 6 for Rotarod; *n* = 7 for adhesive removal). *P < 0.05, 2-way ANOVA repeated measurement.

We initially utilized cytometry by time of flight (CyTOF) to comprehensively assess alterations in peripheral immune cells in aged mice (Fig. 1A). Using specific marker antibodies, we identified the main immune cell subsets, including neutrophils, monocytes/macrophages (MM), T cells, B cells, NK cells, and a minor population of dendritic cells (DC) (Fig. S1A). A decline in the proportion of lymphocytes, specifically T cells, B cells, as well as NK cells, was observed in the aged mice. In contrast, the proportion of myeloid cells increased, with neutrophils showing a particularly notable rise (Fig. 1A). Subsequent SPADE (Spanning-tree Progression Analysis of Density-normalized Events) analysis [17], employing CD11b as a myeloid cell marker, also suggested the expansion of neutrophil clusters (CD11b+Ly6G+) in the peripheral blood of aged mice (Fig. 1B).

To more explicitly understand the impact of aging on peripheral immune cells, as well as how these alterations affect the immune microenvironment following middle cerebral artery occlusion (MCAO), we isolated total blood immune cells from both young and aged mice as well as the CD45hi immune cells in both age groups at 14 d post-MCAO, for subsequent single-cell RNA sequencing (scRNA-seq) (Fig. 1C and Fig. S1B and C). After quality control, 45,649 cells were subjected to downstream analyses. Unsupervised clustering revealed 7 major cell groups: MM, B cells, T cells, neutrophils, NK cells, and 2 DC subsets (cDC and pDC) (Fig. 1C and Fig. S1D) (Table S1). We observed that most immune cell types exhibited varying degrees of infiltration at 14 d post-MCAO. Notably, B cells, which constituted a higher proportion in peripheral blood, were underrepresented in the brain at 14 d post-MCAO. In contrast, MM, which were less prevalent in the periphery, made up a higher proportion of brain-infiltrating immune cells. Additionally, T cells and neutrophils were highly represented among brain-infiltrating cells, suggesting they may play significant roles at this stage (Fig. S1E). Moreover, when comparing the proportional changes in the number of infiltrated immune cells between aged and young mice, we identified an interesting “amplification” phenomenon. Trends observed in peripheral blood cell numbers were magnified in the aged brain. Specifically, aged mice exhibited a higher proportion of neutrophils and MM, and this increase was more pronounced in the brain. Conversely, the proportion of T cells and NK cells declined in peripheral blood in aged mice, and this decline was more marked in the brain (Fig. S1E). We further performed flow cytometry to validate the differences in immune cell populations in the brain. The results indicated that following stroke, the brains of aged mice exhibited an increased infiltration of neutrophils and MM, while the infiltration of NK cells significantly decreased. These findings were consistent with the results obtained from scRNA-seq. We did not observe a significant difference in T cells infiltration between the aged and young groups (Fig. 1D).

From the perspective of cell subset functionalities, we observed a similar “amplification” or correspondence effect with the quantitative change. Specifically, immune cell functions such as antigen presentation, cytotoxicity, and cytokine signaling pathways were markedly heightened in both the aged peripheral blood and brain tissue as compared to their young counterparts (Fig. 1E). Additionally, we observed an up-regulation of antigen presentation capabilities and interferon signaling pathways across all immune cell subsets in the aged group, encompassing both blood and ischemic brain samples (Fig. 1F). Interestingly, these widespread functional alterations were already manifest in the peripheral blood of the aged mice (Fig. S1F). These findings indicate that such changes may represent hallmark features of the aging process.

Subsequently, we further evaluated the impact of aged immune cells on stroke outcomes. Peripheral blood immune cells from either young or aged mice were transferred into the recipient young mice (Fig. 1G). Notably, we found that mice received immune cells from aged donors had larger infarct volumes compared to those received immune cells from young donors (Fig. 1G). Through behavioral assessments, we observed that mice received aged immune cells demonstrated a poorer trend in sensory-motor functional recovery (Fig. 1H and I). Furthermore, these disparities remained evident at day 14, a phase commonly acknowledged as the post-MCAO repair phase. Additionally, this increase in infarct volume and impaired functional recovery were accompanied by a higher infiltration of neutrophils, MM, CD4 T cells, and CD8 T cells, suggesting an intensified inflammatory response (Fig. S1G).

### Peripheral neutrophil senescence intensifies with aging and was amplified in the brain post-stroke

**Fig. 2.**
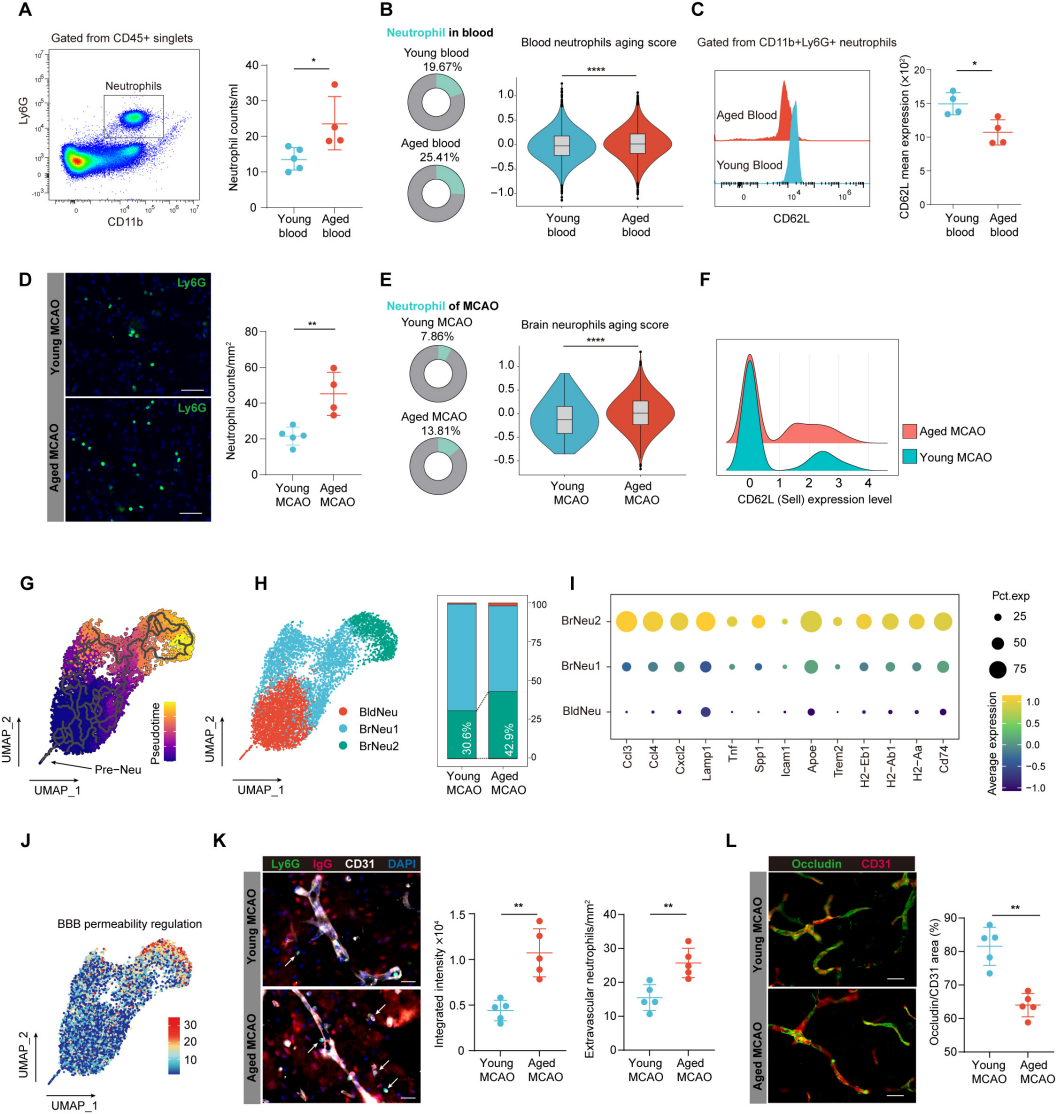
Aged mice exhibit accelerated neutrophil aging. (A) Schematic diagram illustrating the gating strategy for neutrophil quantification using flow cytometry, where neutrophils were identified as CD11b+Ly6G+ cells. Results indicate an increased number of peripheral blood neutrophils in aged mice (*n* = 4) compared to young mice (*n* = 5). *P < 0.05, Student *t* test. (B) Left panel: scRNA-seq indicating that the proportion of neutrophils among immune cells increased in the aged blood. Right panel: Violin plot showing elevated neutrophil aging scores in aged mice. ****P ≤ 0.0001, Bonferroni-corrected Wilcoxon rank sum test. (C) Flow cytometry indicating that aged mice express lower levels of CD62L in peripheral blood neutrophils than young mice, *n* = 4 per group, *P < 0.05, Student *t* test. (D) Immunofluorescence staining showing that at 14 d post-MCAO, aged mice (*n* = 4) had more neutrophil infiltration in the ischemic brain compared to young mice (*n* = 5). Scale bar = 50 μm, **P < 0.01, Student *t* test. (E) scRNA-seq at 14 d post-MCAO suggesting an increased proportion of neutrophils among immune cells in the ischemic brain of aged mice; violin plot showing elevated peripheral neutrophil aging scores in aged mice. ****P ≤ 0.0001, Bonferroni-corrected Wilcoxon rank sum test. (F) Ridge plot indicating down-regulated expression of Sell in neutrophils of aged ischemic brain. (G) Pseudotime trajectory of peripheral and ischemic brain neutrophils analyzed with Monocle3. (H) UMAP plot of peripheral and ischemic brain neutrophils from aged and young mice (left), and stacked bar plot showing the proportion of the 3 clusters (right). (I) BrNeu2 exhibited higher levels of chemotaxis, secretion, and antigen presentation-related genes. (J) Feature plot displaying the BBB permeability regulation score. (K and L) Immunofluorescence staining indicating that at 14 d post-MCAO, aged mice have more extravasation of endogenous IgG and neutrophils (white arrow) (K) as well as lower Occludin/CD31 area ratio (L) in the ischemic brain compared to young mice. *n* = 5 per group. Scale bar = 25 μm. **P < 0.01, Student *t* test. Pre-Neu, neutrophil precursor; BldNeu, peripheral blood neutrophils; BrNeu, neutrophil in the ischemic brain; DAPI, 4′,6-diamidino-2-phenylindole.

As mentioned above, we observed an increased proportion of neutrophils in aged mice. Further, we employed flow cytometry to quantify the neutrophil counts in the peripheral blood of mice. We discovered that aged mice exhibited higher neutrophil counts compared to their young counterparts (Fig. 2A). Utilizing scRNA-seq, we observed not only an increased proportion of neutrophils among peripheral immune cells but also a shift toward a “senescent” phenotype, as indicated by the elevated aging score in neutrophils from aged mice (Fig. 2B). CD62L protein expression was used as a marker to assess neutrophil aging [18]. Upon evaluating the expression levels of this molecule in the peripheral neutrophils of mice, we found that aged mice exhibited lower levels of CD62L expression compared to young mice, suggesting a higher propensity for aging (Fig. 2C). Additionally, scRNA-seq data revealed that, neutrophils from aged mice exhibited diminished Cxcr2 expression but enhanced Cxcr4 expression compared to those from young mice, and other genes associated with advanced neutrophil maturation stages, such as Ifitm1 and Wfdc17 (Fig. S2A), were also up-regulated, suggesting an accelerated senescence process in peripheral neutrophils of aged mice [14,19,20]. In alignment with previous findings [18], we identified an association between neutrophil senescence and elevated inflammatory factor production (Il1b) as well as enhanced vascular injury function (Mmp9) in the peripheral blood neutrophils of aged mice (Fig. S2B). Overall, relative to young mice, aged mice exhibited an increased count of peripheral blood neutrophils that manifested a senescent phenotype. This senescent state predisposed the neutrophils to be more activated, with augmented inflammatory and antigen-presenting capabilities (Fig. S2B).

We then sought to further dissect the maturation stages of these cells in peripheral blood. Using the presence of Pre-Neu, a precursor of neutrophils, as a developmental starting point, we employed Monocle3 [21] to discern the maturation trajectory (Fig. S2C and D). As a result of this comprehensive analysis, we categorically distinguished neutrophils into 3 distinct groups based on their maturity. The first group comprised the aforementioned Pre-Neu, representing the earliest stage of development. Subsequently, 2 mature stages were identified: BldNeu1, indicative of early-stage mature neutrophils, and BldNeu2, representing the later stage of maturation. In addition, we observed an increase in the proportion of BldNeu2 in the peripheral blood of aged mice (Fig. S2E), suggesting an age-related inclination toward the advanced neutrophil maturation phase. Neutrotime depicts a continuous developmental trajectory of neutrophils [22]. We observed that Pre-Neu distinctly expressed genes associated with early neutrotime, while in contrast, BldNeu2 prominently expressed genes characteristic of late neutrotime (Fig. S2F). Interestingly, as a representation of a later neutrophil development state, BldNeu2 demonstrated a higher aging score compared to BldNeu1 and (Fig. S2G). These findings lead us to postulate that neutrophils experience a systematic senescence during their developmental and maturation phases—a process seemingly intensified in the peripheral blood of aged mice. This senescent state of neutrophils is marked by enhanced adhesion, antigen synthesis, increased cytotoxicity, and bolstered immune responses (Fig. S2H).

Subsequently, we investigated alterations in the neutrophils within the aged brain and their relationship with the senescence of peripheral neutrophils. Immunofluorescence staining highlighted that there was a greater infiltration of neutrophils in the brains of aged mice 14 d post-MCAO compared to the young mice (Fig. 2D). Under aged conditions, the proportion of neutrophils among immune cells following MCAO was elevated. Furthermore, paralleling our observations in the periphery, neutrophils in the aged brain exhibited a more pronounced aging phenotype than those in young brains, with this trend being more evident than in peripheral blood (Fig. 2E). The expression of CD62L (encoded by Sell) in neutrophils from the ischemic brain of aged mice was also found to be lower compared to that in young mice (Fig. 2F). We employed Pre-Neu as a starting reference to illustrate the pseudotime distribution of neutrophils from the peripheral blood and those infiltrated in the brain (Fig. 2G). Based on this distribution, we defined Pre-Neu and early-evolutionary stage neutrophils as BldNeu, given that they predominantly originated from peripheral blood. Neutrophils that appeared most mature in the pseudotime developmental trajectory were categorized as BrNeu1, as they were almost exclusively mapped to the ischemic brain. Other neutrophils distributed within the brain were collectively designated as BrNeu2 (Fig. 2H). The above results also suggest that neutrophils infiltrated the brain positioned at a later pseudotime endpoint compared to their peripheral counterparts. We observed that a group of BrNeu1 neutrophils could be mapped to the peripheral blood. These neutrophils exhibited a later developmental state on the pseudotime trajectory compared to other circulating neutrophils, suggesting that they may represent a more senescent population of neutrophils in the blood. Moreover, this subset of neutrophils also showed an increase in the peripheral blood of aged mice (Fig. S2I).

BrNeu2 resided at the furthest endpoint in the pseudotime developmental trajectory (Fig. 2G) and exhibited lower expression levels of Cxcr2 and Sell, suggesting a potential senescent state (Fig. S2J). Moreover, BrNeu2 was more prevalent in the brains of aged mice (Fig. 2H). Compared to other neutrophils, BrNeu2 had elevated expression of cytokine genes and genes associated with neutrophil degranulation (Ccl3, Ccl4, Cxcl2, lamp1, and Tnf), as well as adhesive function-related molecules (Icam1 and Spp1). Intriguingly, BrNeu2 also displayed increased expression genes-related antigen presentation (H2-Eb1, H2-Ab1, H2-Aa, and Cd74) (Fig. 2I). Furthermore, what’s of greater significance is our discovery that BrNeu2 has a substantial impact on the blood–brain barrier (BBB) permeability (Fig. 2J). We then assessed the BBB permeability in aged and young mice 14 d post-MCAO. The results indicated more pronounced extravasation of endogenous immunoglobulin G (IgG) and neutrophils, along with reduced tight junctions in endothelial cells in the brains of aged mice, indicating a more pronounced disruption of the BBB (Fig. 2K and L). Furthermore, vascular coverage in aged mice was lower than in young mice, suggesting impaired vascular repair in the elderly environment (Fig. S2K).

Our findings suggest that, aged mice display more senescence characteristics in peripheral blood neutrophils compared to young mice. This may contribute to the increased senescent neutrophils observed in the brains of aged mice following ischemic stroke. Such senescent neutrophils appear to be more inclined toward a destructive state, potentially exacerbating unfavorable outcomes after stroke.

### Functional shift in MM toward enhanced antigen presentation in aged mice

**Fig. 3.**
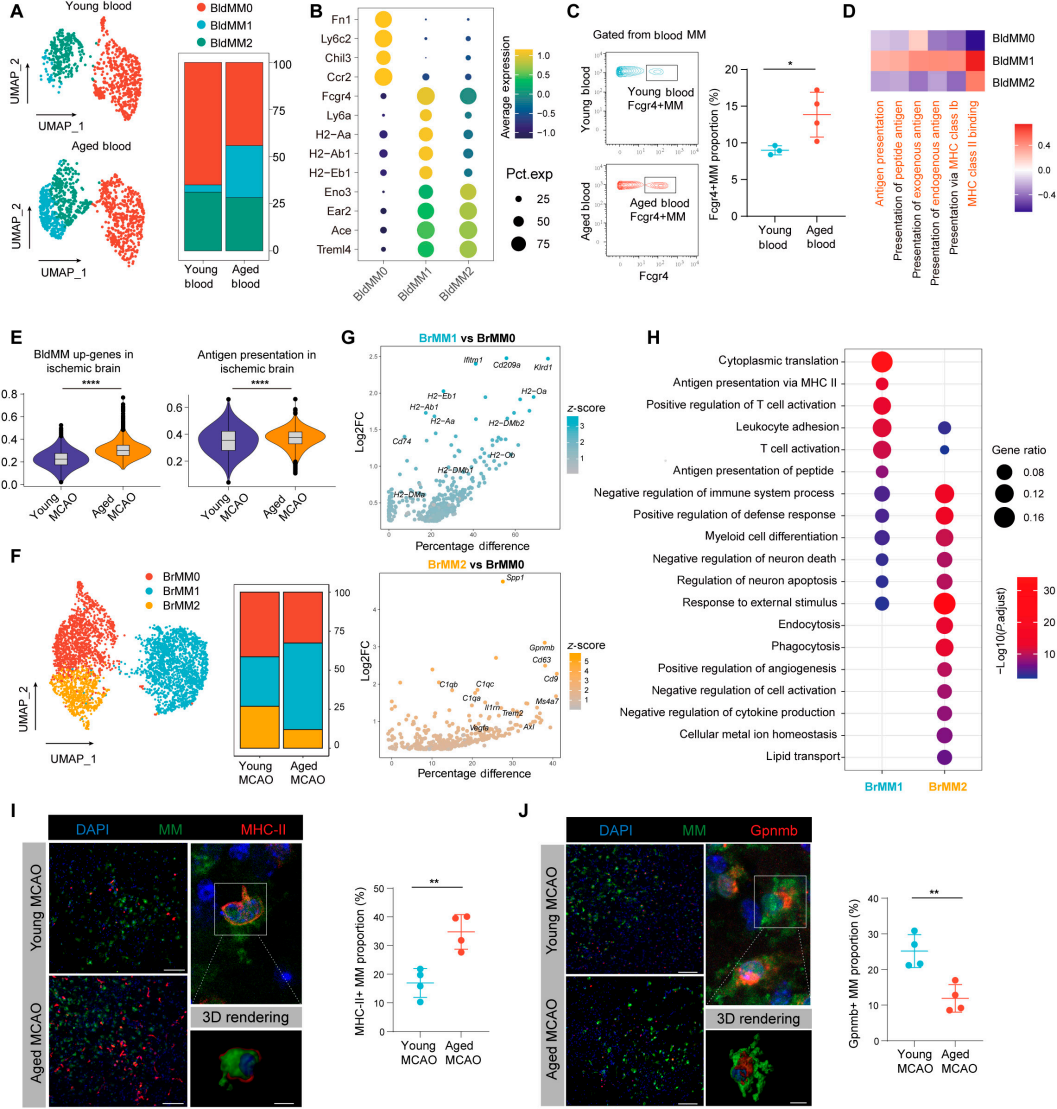
Changes in MM functional states in aging. (A) UMAP plot of peripheral MM from aged and young mice showing increased proportion of BldMM1 in peripheral blood of aged mice. (B) Dot plot of marker genes for the 3 MM subclusters. (C) CyTOF results indicating a higher proportion of Fcgr4+ MM (gated from CD45+CD11b+Ly6g-Ly6c+F4/80+ cells) subgroups in the peripheral blood of aged mice (*n* = 4) compared to young mice (*n* = 3). *P < 0.05. Student *t* test. (D) Enrichment of several antigen-presentation functional pathways in BldMM cell population. (E) Violin plot showing up-regulated genes in peripheral MM of aged mice (left) and antigen presentation function (right) were both elevated in the infiltrated MM of the aged mice compared to those of young mice at 14 d post-MCAO. ****P ≤ 0.0001, Bonferroni-corrected Wilcoxon rank sum test. (F) UMAP visualization showing the distribution and proportions of the 3 distinct MM subsets in the ischemic brain. (G) Scatter plot showing the up-regulated DEGs with a log2(fold change) > 0.25 in BrMM1 (top) or BrMM2 (bottom) compared to BrMM0, with the percentage difference along the x-axis and log2(fold change) along the y-axis. The combined *z*-score of percentage difference and log2(fold change) is shown in the color scale. (H) Dot plot illustrating representative functional terms of BrMM1 and BrMM2 by GO enrichment based on *z*-score and significance (−log10[adjusted *P* value]). (I and J) Quantitative comparison of the numbers of MHC-II+ (I) or Gpnmb+ (J) MM in the ischemic brain between aged and young mice. Scale bar =100 μm. Three-dimensional constructed image showing the morphology of MHC-II+ and Gpnmb+MM. Scale bar = 5 μm. *n* = 4 per group. (I and J), **P < 0.01, Student *t* test. BldMM, peripheral blood monocytes/macrophages; BrMM, monocytes/macrophages in the ischemic brain.

We observed a notable shift in the overall gene expression profile of MM in the peripheral blood. Specifically, there was an enhanced expression of antigen presentation-related genes, while the expression of classical MM genes, such as Ccr2, Ly6c2, and Trem2, was markedly reduced (Fig. S3A). This suggests a functional transition toward antigen presentation of MM in aged mice blood. Three distinct clusters emerged within the blood MM population. BldMM0, indicative of the classical MM characterized by Ccr2 and Ly6c2 expression, having a reduced proportion in aged mice. In contrast, BldMM2, characterized by a diminished Ly6c expression and enriched of nonclassical monocyte markers Eno3, Ear2, and Ace [23], maintained consistent across both age groups. Interestingly, BldMM1, a subset that highly expressing Fcgr4 and Ly6a, scarcely found in young mice but abundant in the aged, demonstrated heightened expression of antigen-presenting genes, including H2-Aa, H2-Ab1, and H2-Eb1 (Fig. 3A and B). The results from CyTOF also confirmed that the proportion of Fcgr4+MM (one of the marker genes for BldMM1) significantly increased in the peripheral blood of aged mice (Fig. 3C). Functional enrichment analysis further revealed that BldMM1, in comparison to other peripheral macrophages, distinctly showcased enhanced antigen-presentation capabilities (Fig. 3D).

Further, we probed if this peripheral MM functional transition influenced the MM’s role in the brain at 14 d post-MCAO. Our findings resonated with our earlier observations: genes up-regulated in peripheral MM of aged mice comparted with young mice, as well as the genes related to antigen-presentation function were up-regulated in MM in the aged mice’s brain (Fig. 3E). In the brain, we identified 3 MM subtypes: BrMM0, the classical MM with a Ly6c+Ccr2hi profile; BrMM1, showing a preference for antigen-presentation genes; and BrMM2, distinguished by Gpnmb and Trem2 markers (Fig. 3F and G and Fig. S3B and C). In alignment with peripheral findings, there was a significant increase of antigen-presentation related BrMM1 (Fig. 3F and H and Fig. S3D). In contrast, BrMM2, representing a distinct subset of MM known for its pronounced phagocytic and reparative capabilities, exhibited a reduced presence in the aged brain (Fig. 3F and H).

Finally, performing immunofluorescence staining, we observed an increase in the number of antigen-presenting MMs in aged brains (Fig. 3I). Conversely, the quantity of BrMM2 (Gpnmb+MM) notably decreased (Fig. 3J).

### Altered T cell composition and increased T cell activation with aging

**Fig. 4.**
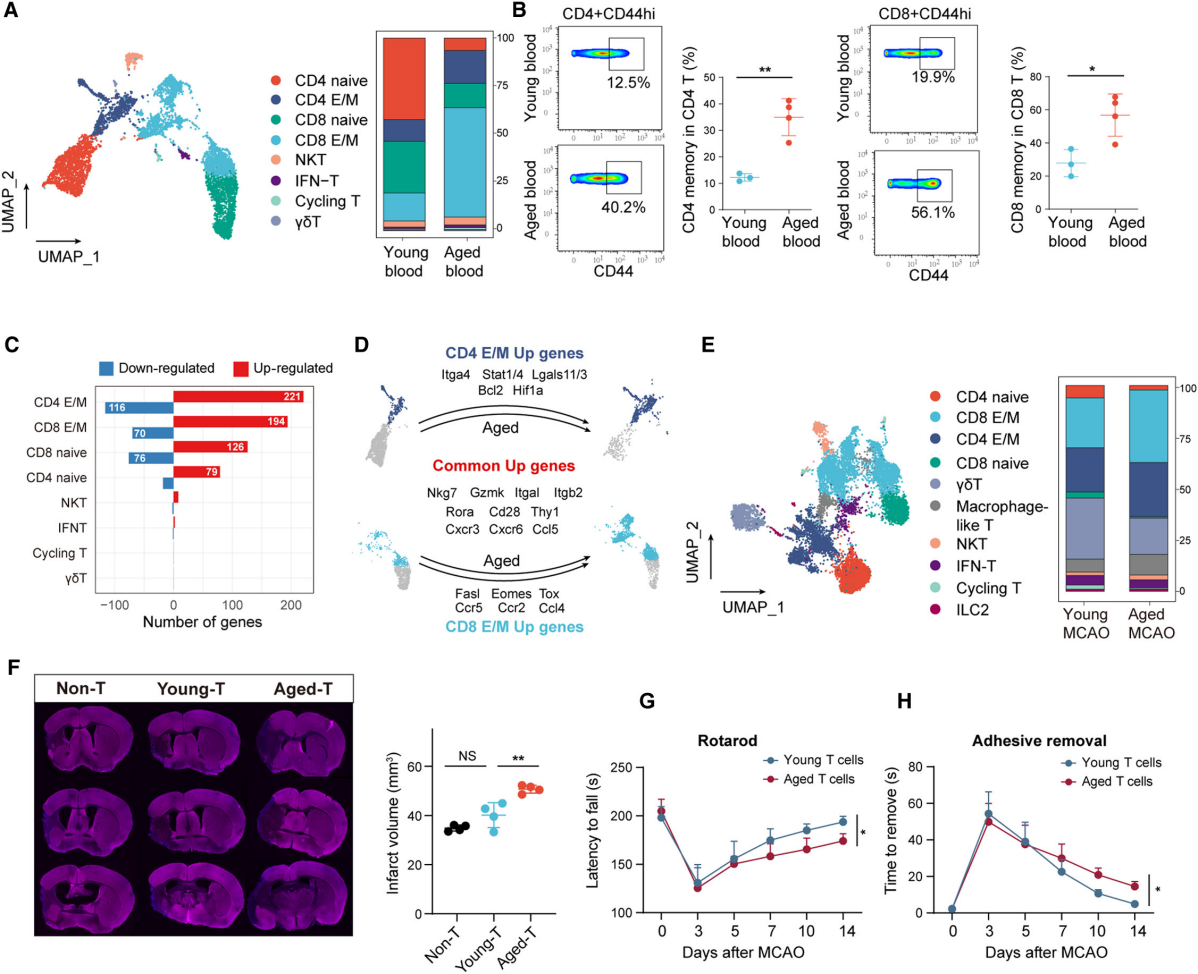
Altered subgroup composition in T cells due to aging. (A) UMAP plot of peripheral T cells from aged and young mice (left). Bar plots showing the proportions of the different T cell subsets (right). (B) CyTOF results indicating a significant increase in the proportion of memory cells expressing CD44hi of CD3+CD4+T and CD3+CD8+T cell populations in aged mice (*n* = 4) compared to young mice (*n* = 3). *P < 0.05, **P < 0.01, Student *t* test. (C) Bar graph showing the number of DEGs in each subgroup in the peripheral blood of aged and young mice, with a log2(fold change) > 0.25 and adjusted *P* value < 0.05. (D) Display of genes up-regulated in CD4 E/M and CD8 E/M cells in aged mice compared to young mice. (E) UMAP plot of peripheral and brain T cells from aged and young mice (left), along with the proportion of these T cell clusters in aged and young ischemic brains (right). (F) Mice that received aged T cell showing larger infarct volumes at 14 d post-MCAO. *n* = 4 per group. **P < 0.01, Student *t* test. NS means not significant. (G and H) Rotarod (G, *n* = 7 per group) and adhesive removal tests (H, *n* = 8 per group) suggest that mice receiving aged T cells exhibit worse neurological outcomes than those receiving young T cells. *P < 0.05, 2-way ANOVA repeated measurement. IFN-T, T cells that respond to interferons; Non-T, MCAO mice did not receive T cells; Young-T, MCAO mice received young T cells; Aged-T, MCAO mice received aged T cells.

T cells play a pivotal role in adaptive immunity. In the peripheral blood, based on characteristic marker genes and cell surface molecules (Fig. S4A), namely Cd4, Cd8, sell, ccr7, cd44, and klrb1c, we delineated various subsets of T cells. These subsets include CD4 naive, CD4 effector/memory (CD4 E/M), CD8 naive, CD8 effector/memory (CD8 E/M), and NKT cells. Additionally, the peripheral blood comprises a minority of T cells that respond to interferons, termed IFN-T cells, characterized by their expression of genes such as Ifit1, Ifit3, and Isg15. We also identified cycling T cells, marked by Mki67, Ccnb2, Cdca3, and Ccna2, as well as γδT cells, indicated by the presence of Trdv4 and Trdc genes (Fig. 4A and Fig. S4A and B). Among these cells, we observed a significant decline in the proportions of both CD4 naive and CD8 naive T cells in the peripheral blood of aged mice. Conversely, CD4 E/M and CD8 E/M, exhibited a pronounced increase in their numbers (Fig. 4A). Flow cytometry analyses also confirmed an increase in the proportion of CD4 memory and CD8 memory (Fig. 4B).

Subsequently, we delved deeper into examining the gene expression differences of each T cell subset between the aged and young mice. The findings indicated that CD4 E/M and CD8 E/M displayed the most pronounced differences. For other T cell subsets, including NKT, we did not observe significant gene expression variations (Fig. 4C). CD4 E/M cells exhibited significant up-regulation of genes related to adhesion, differentiation, and secretory factor functions (such as Itga4, Stat1/4, and lgals1/3) as well as T cell survival associated genes (Bcl2 and Hif1a). In contrast, CD8 E/M cells displayed prominent up-regulation of genes associated with cytotoxicity, exhaustion (like Fasl, Eomes, and Tox), and chemotactic functions (such as Ccr5, Ccr2, and Ccl4). Furthermore, several genes were up-regulated in both cell types, with their functions relating to cytokine release, cytotoxicity, T cell adhesion, and T cell proliferation, reflecting attributes of activated T cells (Fig. 4D and Fig. S4C). We named these genes that up-regulated in both CD4 E/M and CD8 E/M as common up genes (Table S2).

Integrating our observations from the peripheral blood T cells with those from the brain post-MCAO, several T clusters were identified (Fig. 4E and Fig. S4D). The increase in the proportion of E/M cells with aging, as seen in the periphery, was consistently reflected post-stroke in the aged brain (Fig. 4E). We then assessed the expression of the common up genes within infiltrated T cells and found that these genes were predominantly expressed by CD4 E/M and CD8 E/M in the brain. Interestingly, while these up-regulated genes were primarily concentrated in these subsets, almost all of T cell groups in the aged brain demonstrated an increased expression of these genes compared to their young counterparts (Fig. S4E and F). This observation further underscores the pervasive influence of aging on shaping the functional landscape of T cells.

Finally, to verify the impact of aged peripheral T cells on the prognosis of MCAO in mice, we isolated T cells from aged or young mice and transferred them into young mice, which were subsequently subjected to MCAO. The outcomes revealed that mice received young T cells did not result in a significant change in infarct volume (Fig. 4F). However, mice received aged T cells demonstrated larger infarct volumes by day 14 post-MCAO and exhibited poorer neurological function recovery (Fig. 4G and H). This indicates a detrimental role of aged T cells in stroke outcomes, underscoring the importance of understanding the age-related changes in immune cell functionality and their implications in neurological diseases.

### Age-associated shifts in CD4 T cell subtypes in peripheral blood and ischemic brain

**Fig. 5.**
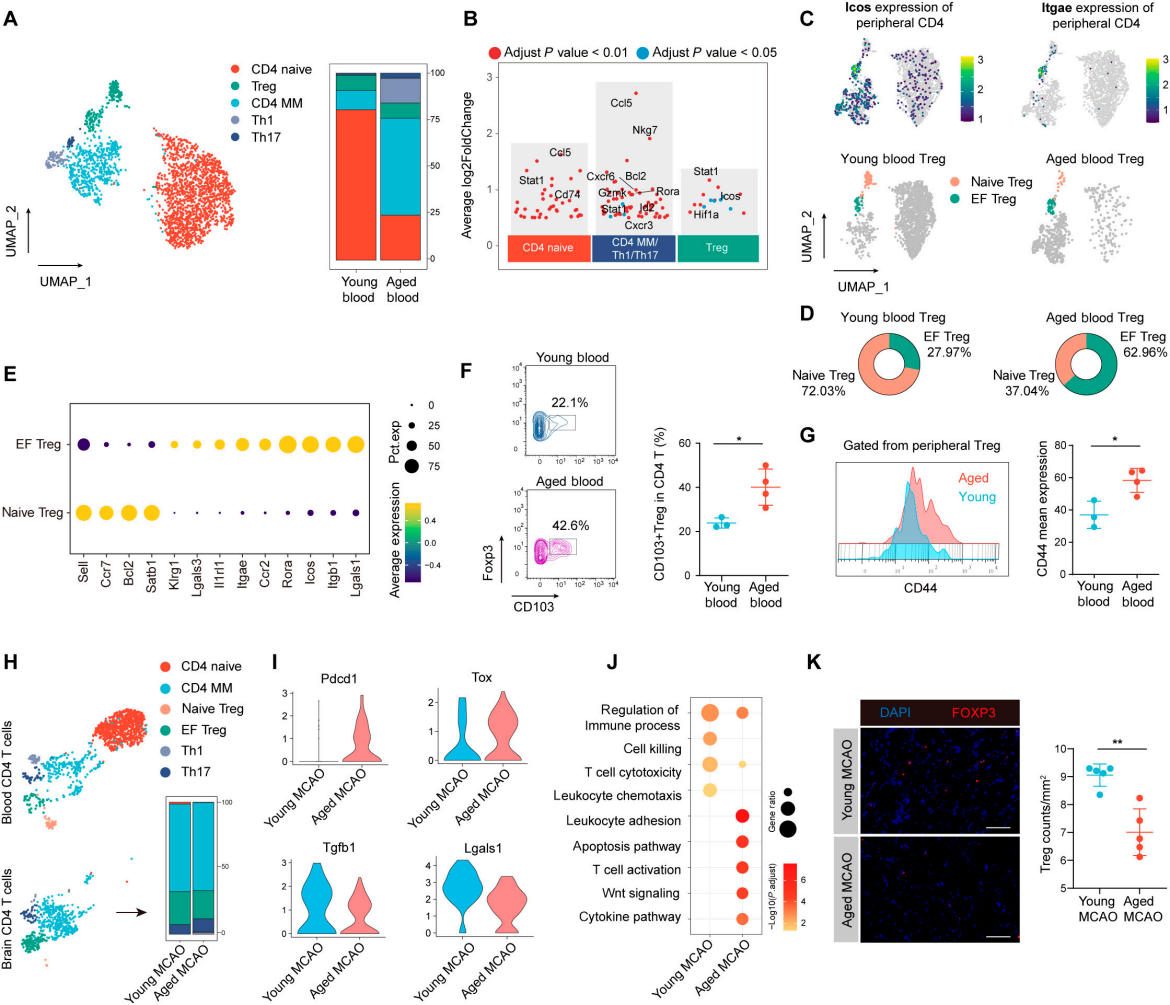
Functional changes in CD4 T cells in aged mice. (A) Cell clustering of peripheral blood cells from aged and young mice (left), and differences in the proportions of cell subsets between the 2 groups (right). (B) Scatter plot showing differential genes in 3 major categories of peripheral blood CD4 T cells in aged versus young mice: The y-axis displays the log2(fold change). (C) Upper panel displaying the gene expression distribution of Icos and Itgae; lower panel showing the UMAP of naive Tregs and EF Tregs. (D) The proportion of EF Tregs in total Tregs was higher in aged mice compared to young mice. (E) Dot plot showing the expression of marker genes in naive Tregs and EF Tregs. (F and G) CyTOF data showing that the proportion of peripheral blood CD103+ Tregs was higher in aged mice (*n* = 4) compared to young mice (*n* = 3) (F), and that the expression level of CD44 of Tregs was elevated in aged mice (*n* = 4) compared to young mice (*n* = 3) (G). Tregs were gated as CD3+CD4+CD25+FOXP3+cells. *P < 0.05, Student *t* test. (H) UMAP projection plot showing cell clusters of peripheral and infiltrated CD4 T cell clusters from aged and young mice. A stacked bar plot in the right-bottom panel showing the proportions of these clusters within the ischemic brains of aged and young mice. (I) Violin plot showing differences in the expression of Pdcd1, Tox, Tgfb1, and Lgals1 in EF Tregs in the ischemic brains of aged and young mice. (J) GO enrichment analysis showing functional differences between aged and young mice. (K) Immunostaining results indicating that at 14 d post-MCAO, aged mice have fewer Tregs counts in the ischemic brain compared to young mice. *n* = 5 per group. Scale bars = 50 μm. **P < 0.01, Student *t* test. Treg, regulatory T cells; CD4MM, CD4 memory; Th1, T helper 1; Th17, T helper 17.

Next, we conducted a detailed exploration of CD4 T cells. Broadly, based on distinct marker genes, we categorized peripheral blood CD4 T cells into several subtypes: CD4 naive (Sell and Ccr7), CD4 memory (CD4 MM) (Cd44), T helper 1 (Th1) cells (Ifng, Ccr5, and Cxcr6), T helper 17 (Th17) cells (Ccr6 and Ccr4), and regulatory T cells (Tregs) (Foxp3 and Il2ra) (Fig. 5A and Fig. S5A and B). Quantitatively, we found CD4 naive cells decreased while CD4 MM increased in aged mice. Additionally, we noted a significant surge of the Th1 cell population and a slight increase of the Th17 cell subset (Fig. 5A). Functionally, the CD4 subgroups with effector/memory attributes, including CD4 MM, Th1, and Th17, exhibited an up-regulation of the most differential genes compared the others in the peripheral blood of aged mice and demonstrated elevated expression of genes related to effector and chemotactic functions (Fig. 5A and B). This indicates a significant activation of CD4 T cells in the peripheral blood of aged mice.

In the peripheral blood of aged mice, there was an up-regulation of Icos expression in Tregs. Icos+ Tregs are considered functional Tregs [24]. Thus, the increased expression of Icos might indicate a shift of Tregs toward an activated or effector state. We then further refined the categorization of Tregs. Remarkably, Tregs were subdivided based on markers of naive T cells (Ccr7 and Sell) and genes associated with activated Tregs (Icos and Itage), resulting in the classification of 2 subsets: naive Tregs and effector Tregs (EF Tregs). Particularly noteworthy, Itgae expression was exclusively detected in the EF Tregs subset (Fig. 5C and E and Fig. S5A). Overall, EF Tregs exhibited higher expression of genes related to adhesion, chemotaxis, and secretory functions than naive Tregs (Fig. 5E). Pseudotime analysis suggested that naive Tregs share similarities with CD4 naive cells, displaying an earlier differentiation state. In contrast, EF Tregs clearly clustered toward the later end of differentiation (Fig. S5C). After applying this classification to peripheral blood CD4 T cells, we observed a decline in the proportion of naive Tregs in aged CD4 T cells, while the number of EF Tregs increased (Fig. S5D). In terms of the composition within the Tregs population itself, the proportion of EF Tregs in aged mice was approximately 3 times that in young mice (Fig. 5D). We also found that naive Tregs in aged mice exhibited elevated expression of marker genes for EF Tregs (Icos, Itgae, Rora) (Fig. S5E). This indicates that even though the naive Tregs in the peripheral blood of aged mice maintain an overall naive phenotype, they still exhibit a higher level of activation compared to the young mice. By using CyTOF, we observed a higher proportion of CD103 (encoded by Itgae) and increased CD44 expression in peripheral blood Tregs of aged mice (Fig. 5F and G). In addition, differential expression of transcription factor genes among various subgroups of peripheral blood CD4 T cells suggests these transcription factors may contribute to the significant increase in Th1 cells and the activation of Tregs observed in aged mice (Fig. S5F).

Subsequently, we analyzed the changes exhibited by CD4 T cells upon infiltration into the brain. Overall, the infiltrated CD4 T cells were primarily constituted of CD4 MM, Th17, and EF Tregs. We observed an increased proportion of Th17 cells in the brains of aged mice. Conversely, the proportion of EF Tregs declined in aged mice (Fig. 5H). Further investigations into the differences between EF Tregs in aged and young brains revealed that the aged brain’s EF Tregs significantly up-regulated the expression of Pdcd1, which encodes programmed cell death protein 1 (PD-1), and Tox, potentially indicating a shift toward an exhausted state. In line with this, the classical Tregs-produced cytokine genes Tgfb1 and Lgals1, which encodes Galectin-1, one of the mediators of Treg’s immunosuppressive function [25], were down-regulated in Tregs in the aged brain (Fig. 5I). Enrichment analysis revealed that the infiltrated EF Tregs in aged mice showed an increased up-regulation of pathways related to apoptosis, responses to interferon, and oxidative stress compared to their young counterparts. This might partly explain the reduced quantity of EF Tregs in the aged brain (Fig. 5J). By using flow cytometry, we observed that at the protein level, Tregs in the ischemic brains of aged mice expressed higher levels of PD-1 and lower levels of transforming growth factor–β1 (TGF-β1) compared to young mice. This finding was consistent with the scRNA-seq results (Fig. S5G). Additionally, immunofluorescence assays also confirmed lower numbers of Tregs in the brains of aged mice at 14 d post-MCAO (Fig. 5K).

In conclusion, based on our findings, there was a significant increase in the ratio of Th1 cells and Th17 cells in the peripheral blood with aging. Moreover, Tregs were found to be in a more activated state in aged peripheral blood while exhibited a functional exhaustion-like phenotype in the aged ischemic brain.

### Dysregulated immunity with aging: Decline of CD8 Tregs and surge of CD8 EXL in peripheral blood and brain

**Fig. 6.**
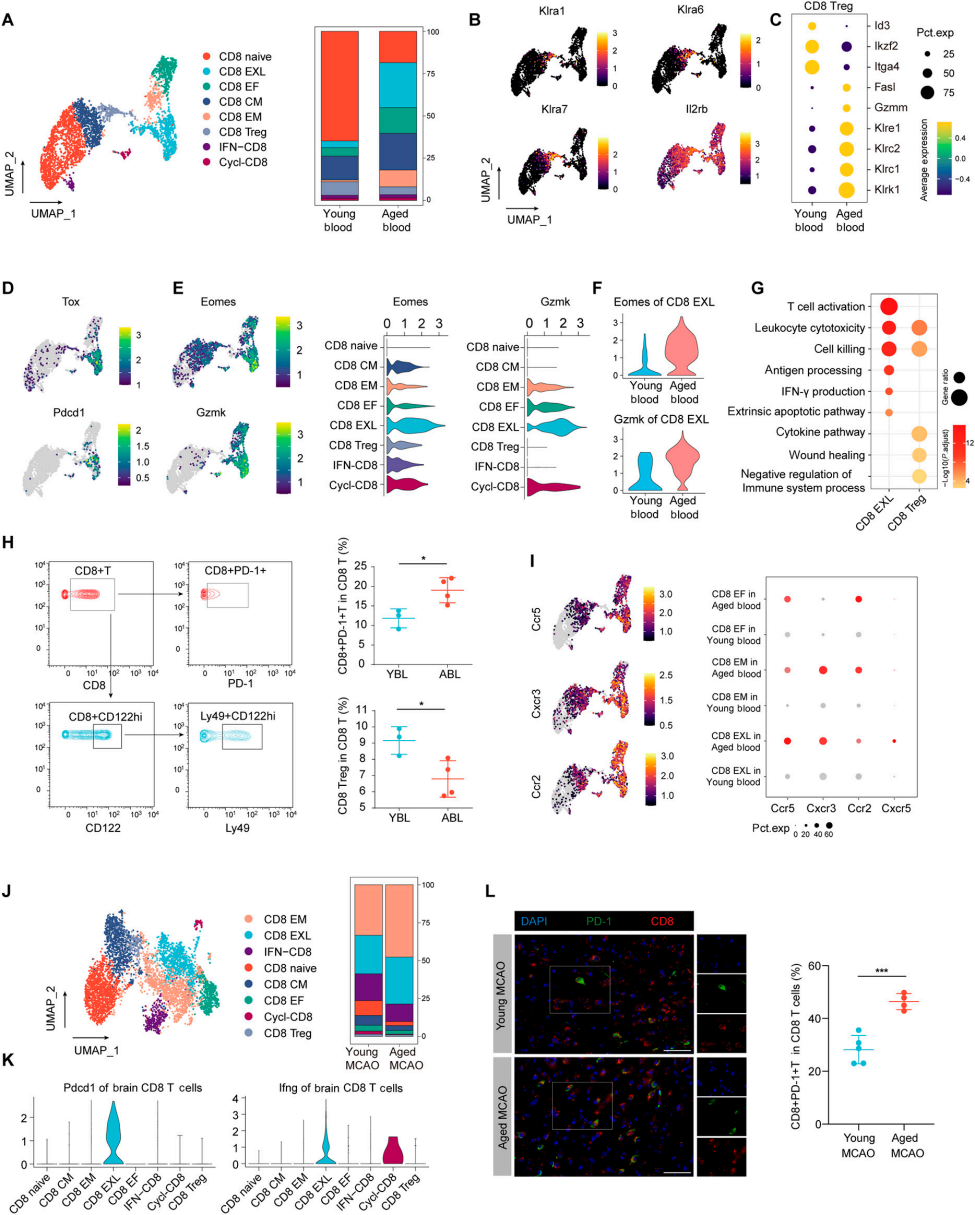
CD8 immune imbalance in aged mice. (A) UMAP plot of all peripheral CD8 clusters from aged and young mice (left), and a stacked bar plot showing the proportion of these clusters in the 2 groups (right). (B) Feature plot illustrating the expression of Klra1, Klra6, Klra7, and Il2rb genes. (C) Dot plot showing different genes expression in CD8 Tregs between aged and young mice. (D) Tox and Pdcd1 were highly expressed in CD8 EXL. (E) Left panel displaying a feature plot of Eomes and Gzmk expression distribution; right panels showing violin plots indicating higher Eomes and Gzmk expression in CD8 EXL. (F) Peripheral blood CD8 EXL in aged mice express higher levels of Eomes and Gzmk compared to young mice. (G) Dot plot illustrating distinct functional terms in CD8 EXL and CD8 Tregs, as revealed by GO enrichment analysis. (H) Gating scheme for CD8+PD-1+T cells and CD8 Tregs of CyTOF analysis (left); results show an increased proportion of peripheral CD8+PD-1+T cells and a significant decrease in CD8 Tregs proportion in aged mice (*n* = 4) compared to young mice (*n* = 3). *P < 0.05, Student *t* test. (I) Left panel showing the distribution of major chemotactic receptors in peripheral CD8 T cells. Right panel: Dot plot indicating that higher expression levels of chemotactic receptors in CD8 EF, CD8 EM, and CD8 EXL in aged mice compared to young mice. (J) UMAP plots of CD8 cell clusters in the peripheral blood and ischemic brain (left). Differences in the proportions of these clusters in the ischemic brain (right). (K) Violin plots indicating that brain CD8 EXL express higher levels of Pdcd1 and Ifng compared to other cell clusters. (L) Immunostaining showing that at 14 d post-MCAO, the proportion of CD8+PD1+T cells in the ischemic brain of aged mice (*n* = 4) was higher than young mice (*n* = 5). Scale bar = 50 μm. ***P < 0.001, Student *t* test. CD8 CM, CD8 central memory; CD8 EM, CD8 effector memory; CD8 EF, CD8 effector; CD8 Treg, regulatory CD8 T cells; IFN-CD8, CD8 T cells that respond to interferons; Cycl-CD8, cycling CD8 T cells.

Our findings revealed a significant decline in the number of naive CD8 T cells in aged peripheral blood. In contrast, we observed an increase in CD8 central memory (CD8 CM) (Ccr7hiCD44hi), CD8 effector memory (CD8 EM) (Ccr7loCD44hi), and CD8 effector (CD8 EF) (Klrg1+Gzma+Gzmb+). Two populations with lower proportions: interferon-responsive CD8 (IFN-CD8) (Ifit1, Ifit3, and Isg15) and cycling CD8 (Cycl-CD8) (Ccna2, Ccnb2, and Mki67) were also identified (Fig. 6A and Fig. S6A). We found a unique subset of CD8 T cells, which was notably diminished in aged mice, characterized by the signature marker molecules Klra1, Klra6, and Klra7 (encoding Ly49A, Ly49F, and Ly49G respectively) (Fig. 6B). Based on previous reports, we designated this group as regulatory CD8 T cells (CD8 Tregs) [26]. In addition, these cells exhibited high expression of Il2rb (CD122) and the transcription factor Ikzf2 (Helios) (Fig. 6B and Fig. S6A), which has been reported as a key molecule in sustaining the suppressive function of regulatory T cells, encompassing both CD4 Tregs and CD8 Tregs [27]. In both aged and young mice, there was no significant alterations in the immunoregulatory capacities of peripheral blood CD8 Tregs (Fig. S6B). However, we observed that the CD8 Tregs in aged mice significantly down-regulated the expression of the Id3 gene, associated with T cell survival and differentiation, and the adhesion-related Itga4 gene. Importantly, the expression of Ikzf2 was also diminished in the aged group. In contrast, aged CD8 Tregs markedly up-regulated genes associated with cytotoxicity and killing functions (Fig. 6C). These findings suggest that CD8 Tregs may exhibit a degree of plasticity and undergo functional shifts in the context of an aging environment.

Another significant alteration in aged mice was the evident increase in the number of exhausted-like CD8 (CD8 EXL) cells (Fig. 6A). CD8 EXL prominently expressed genes encoding inhibitory receptors such as Pdcd1, Lag3, Ctla4, and Cd160 (Fig. 6D and Fig. S6A). They also exhibited high expression levels of Tox and Tcf7 (encoding TCF-1) (Fig. 6D and Fig. S6C). The expression of these 2 genes may suggests that CD8 EXL retains robust functional characteristics [28,29]. Additionally, CD8 EXL highly expressed Gzmk and Eomes (Fig. 6E). Eomes is an essential transcription factor for CD8 T cell differentiation and activation [30–32], and Gzmk+ CD8 cells have been reported to be critical proinflammatory and cytotoxic cells [33–36]. Additionally, we observed that the expression levels of Gzmk, Eomes in CD8 EXL cells were higher in aged mice compared to young counterparts, suggesting a heightened degree of functional activation (Fig. 6F). In summary, the CD8 EXL population, which notably increased with aging, manifested some features of exhaustion genes but still maintained a high degree of cytotoxicity and inflammatory function. Functional enrichment analysis revealed different roles for CD8 EXL and CD8 Tregs. Specifically, CD8 EXL displayed a heightened activation state, with cytotoxic capabilities and IFN production function, while CD8 Tregs demonstrated functions related to the suppression of immune responses (Fig. 6G). The CyTOF analysis corroborated these findings of scRNA-seq: the proportion of CD8 Tregs declined in the peripheral blood of aged mice, while the proportion of CD8 EXL cells increased (Fig. 6H).

We further examined the expression of chemokine receptors in the CD8 EF, CD8 EM, and CD8 EXL, 3 clusters of cells with an increased proportion in the aged peripheral blood compared to young. Cxcr3 was specifically up-regulated in CD8 EM and CD8 EXL, while Ccr2 expression was more noticeably increased in CD8 EM and CD8 EF (Fig. 6I). Interestingly, Cxcr5 expression was relatively unique, being primarily concentrated in the CD8 EXL. Furthermore, the expression of Cxcr5 in CD8 EXL was elevated in aged mice (Fig. 6I and Fig. S6D). These findings suggest that, in addition to the increase in cell numbers, these subsets also exhibit heightened potential for chemotaxis in the aged environment, sharing some common traits in this regard.

Upon infiltrating the brains of MCAO mice, the major CD8 T cell subsets consisted of CD8 EM, CD8 EXL, and IFN-CD8. Notably, the proportions of CD8 EM and CD8 EXL cells were significantly elevated in aged mice (Fig. 6J). In line with our peripheral results, infiltrated CD8 EXL cells, despite exhibiting high expression of exhaustion markers such as Pdcd1, still maintained robust expression of Ifng (Fig. 6K). In addition, CD8 EXL cells expressed genes representing CD8 differentiation or cytotoxicity, such as Tox, Gzmk, and Gzmb, along with cytokine genes like Ccl3, Ccl4, and Il1b (Fig. S6E). Importantly, we found that the infiltrated CD8 EXL cells in aged mice exhibited higher levels of Ccl3, Ccl4, and Il1b expression, suggesting an enhanced proinflammatory function in the aged brain (Fig. S6F). Immunofluorescence confirmed an elevated number of CD8+PD-1+ cells in the brain (Fig. 6L). Interestingly, similar with the results in peripheral blood, we noticed that the aged ischemic brain had fewer CD8 Tregs compared to their young counterparts (Fig. 6J). This finding was further corroborated through flow cytometry analysis. This suggests that the phenomenon of immune imbalance among CD8 T cells was more pronounced in the aged stroke mice (Fig. S6G).

## Discussion

Accompanying the aging process, there is an age-related increase in the levels of inflammation in peripheral blood and tissues, leading to low-grade chronic damage, a phenomenon termed “inflammaging” [37,38]. The immune system plays an integral role in this process, resulting in a state known as immunosenescence [39]. Our current comprehensive study reveals key alterations in the peripheral immune system of aged mice, which can be summarized as follows: (a) Aged mice exhibited an increase in the number of neutrophils in the peripheral blood, and these cells generally displayed a senescent phenotype. (b) The antigen-presenting function of MM was significantly enhanced in the peripheral blood of aged mice. (c) T cells were notably activated in the aged state. Specifically, within the CD4 T cell population, Tregs displayed an effector phenotype, and the proportions of Th1 and Th17 cells were increased. Among CD8 T cells, there was evidence of immune dysregulation: the number of CD8 Tregs was reduced, while the proportion of CD8 EXL cells was significantly elevated. Moreover, these CD8 cells maintained a strong inflammatory and cytotoxic function.

These cellular alterations constituted key aspects of the aged peripheral immune landscape, suggesting a heightened state of immune activation in aged mice compared to young counterparts. Importantly, when considering the changes in the brain’s immune microenvironment at the 14-d repair phase post-MCAO, we observed that the immune aging seen in the peripheral blood experiences an “amplification” effect in the brain, ultimately contributing to poorer outcomes in ischemic stroke in aged mice.

Currently, immunotherapy has emerged as an effective treatment strategy for a wide range of diseases [40]. The field of cellular immune regulation in the context of stroke has also witnessed substantial advancements [41,42]. It is worth noting that three-quarters of stroke occur in people 65 years or older [43]. In this context, it holds great significance to investigate the influence of age-related immune cell alterations on the pathological mechanisms of stroke. Our study sheds light on the alterations in neutrophils, macrophages, and T cells in the context of immune senescence. The immune modulation of these cells may pave the way for future advancements in stroke treatment and prevention.

Neutrophils are among the first cells to respond to injury. Contrary to neutrophils that are recruited into tissues under pathological conditions and subsequently die, neutrophils circulating under normal physiological states do not undergo apoptosis in the bloodstream but are instead cleared within the bone marrow [44]. Previous literature had categorized neutrophils as short-lived cells, with their half-lives in both humans and mice typically measured in hours [45]. However, subsequent research has shown that the life span of neutrophils can be extended under conditions such as inflammation or infection [46]. This phenomenon, currently termed “neutrophil senescence” is characterized by reduced expression of CD62L [18] and elevated expression of CXCR4 [45]. Senescent or aged neutrophils exhibit a heightened state of functional activation, and their proinflammatory activities are positively correlated with their aging status in circulation [20]. In addition, the destructive capabilities of neutrophils toward vasculature also increase with aging [18]. Furthermore, aged neutrophils in the circulatory system arrive more rapidly at the site of inflammation compared to their less-aged counterparts and display stronger chemotactic functions [47]. Our current findings indicate that neutrophils in aged mice exhibit characteristics of senescence, which may contribute to the increased neutrophil count observed in older age. Similarly, we also found that along with this aging, neutrophils in elderly mice display enhanced proinflammatory functions, contributing to the chronic, low-grade inflammation, commonly referred to as “inflammaging,” observed persistently in older individuals.

Gullotta et al.’s research [14] indicates that at 24 h post-MCAO, elderly mice have peripheral blood neutrophils that display more pronounced aging characteristics than their young counterparts. This ultimately leads to poorer reperfusion and prognosis. Our study supports this conclusion and expands these findings across a longer timeframe. Upon integrating scRNA-seq results at 14 d post-MCAO, we observed that neutrophils—the acutely responsive immune cells—were still present in the brain at higher proportions during the chronic phase of stroke. Moreover, their proportion in aged mice at 14 d post-MCAO significantly increased compared to young mice, and these neutrophils also exhibited a heightened state of aging and functional activation within the older brain. Importantly, the phenomenon of neutrophil aging, already present under physiological conditions in elderly mice, was further amplified within the brain. It suggests that intervening in neutrophil senescence at the basal state in aged mice could be a strategy to preemptively alleviate inflammatory damage in diseases such as ischemic stroke.

We observed significantly enhanced antigen presentation capabilities across nearly all peripheral immune cells in aged mice, suggesting that this could be a hallmark of immunosenescence. The role of MM as antigen-presenting cells during aging has garnered divergent views in prior research. While some studies suggest that the antigen-presentation abilities of MM are unaltered with aging [48], others have reported compromised phagocytic functions and impaired antigen presentation in aged mouse MM [49,50]. Conversely, some work indicates an uptick in antigen presentation capabilities, accompanied by an increase in the population of major histocompatibility complex (MHC)-expressing MM in older mice [51,52]. Our results align with this latter perspective, showing an elevated proportion of MM with antigen-presenting capabilities in the peripheral blood of aged mice, thereby resulting in a notable amplification of overall antigen-presentation functionality.

At present, there is insufficient evidence to fully account for the discrepancies observed across these studies. These variations may be attributed to differences in the age of human subjects or the months of age in mice, as well as the diverse methodologies employed, such as in vivo versus in vitro experiments. For instance, in a seminal study examining the effect of aging on macrophage antigen-presenting functions, the authors used bone marrow-derived macrophages cultured in vitro and found that these cells from aged mice displayed lower expression of MHC-II molecules [50]. On the other hand, Barman and colleagues [52], in line with our present research, directly assessed the differences in MM in the peripheral blood between young and aged mice, concluding that the latter had an increased number of antigen-presenting MM. Notably, the mice used in their study were of an even more advanced age. In summary, given the crucial role of MM as antigen-presenting cells, the alterations in their functionalities within an aging environment warrant further investigation and validation. Understanding the modulation of these antigen-presenting capabilities holds significant implications for the functional regulation of other immune cells, particularly T cells.

Despite these discrepancies, many studies related to MM antigen-presentation functionalities have cited the role of interferons. In fact, interferons, particularly IFN-γ, have been extensively investigated for their regulatory effects on MM. Numerous studies have found that interferons can induce MM polarization, thereby enhancing their various functions, including antigen presentation [53–55]. Consistent with these findings, we also observed an up-regulation of genes related to the interferon pathway in MM from peripheral blood of aged mice, which could be linked to the enhancement of their antigen-presenting capabilities.

It is worth mentioning that MM are immune cells with significant plasticity. Previous research has often utilized the concept of “polarization” to categorize MM, where M1-type MM generally exhibit proinflammatory properties and M2-type MM possess strong phagocytic and tissue repair functions [56,57]. In our study, we did not identify a significant peripheral subpopulation of MM with repair capabilities. However, 14 d after MCAO, we discovered a distinct MM subgroup, which we termed BrMM2, characterized by the expression of the Gpnmb gene. BrMM2 shows potential for promoting angiogenesis as well as strong phagocytic activity. Importantly, the number of Gpnmb+ MM in the brains of aged mice during the reparative phase was fewer compared to young counterparts, which may contribute to worse functional outcomes.

There has been extensive research on the effects of aging on T cells, and it is widely accepted that there is a naive–memory imbalance in aged individuals, characterized by a reduction in naive T cells and an increase in memory T cells [58,59]. Our current study corroborates this phenomenon at both the scRNA-seq and protein expression levels. Additionally, we identified several genes that are up-regulated in the T cells of aged mice, suggesting that these genes could be important targets for intervening in immunosenescence.

Regulatory T cells are typically referred to as CD4 Tregs. Interestingly, we found that both in aged and young peripheral blood, Tregs could further be classified into naive Tregs and EF Tregs. No significant transcriptional changes were observed in EF Tregs between the 2 age groups. Instead, the functional disparities among Tregs were mainly concentrated in naive Tregs, manifesting as heightened activation and effector status. Upon recruitment to the ischemic brain region, not only were there fewer Tregs in aged mice, but these cells also exhibited higher expression of PD-1 and reduced expression of immunosuppressive molecules. Since aging did not significantly affect the gene expression of EF Tregs, including genes encoding chemokine receptors, the observed reduction in the number of Tregs in the aged brain may not be a result of differences in their chemotactic abilities. Our findings revealed that Tregs in the aged brain exhibit a higher level of apoptotic activity. Interestingly, previous research indicating that Tregs in tumor microenvironments tend to undergo apoptosis in response to elevated levels of reactive oxygen species (ROS) [60]. In addition, some studies have suggested that the concentration of ROS in the aged stroke brain is higher than in younger brains [61]. Further investigation is warranted to determine whether the decrease in the number of Tregs in aged stroke is related to changes in ROS or other cytokines in the aging brain tissue. PD-1 is a critical marker of T cell exhaustion, which usually arises due to prolonged antigen exposure [62–64]. As described earlier, the increased antigen-presenting capabilities of various immune cells in the aged environment leads us to speculate that peripheral Tregs in aged mice may already be in a state of overactivation. Moreover, the exacerbated inflammatory environment post-MCAO in aged mice further contributes to the exhaustion of these cells upon entering the brain. Previous studies have indeed reported weakened immunosuppressive functions of PD-1+ Tregs [65], and it has been shown that the immunosuppressive ability of these Tregs can be restored through PD-1 blockade [65,66].

In our study, we reported a distinct subset of CD8 T cells characterized by the expression of genes encoding Ly49 markers (Klra1, Klra6, Klra7), along with high levels of CD122 and Helios. As mentioned earlier, Helios is a critical transcription factor in regulatory T cells for exerting immunosuppressive functions. This population of CD8 T cells shares molecular characteristics with previously reported immunosuppressive CD8 Tregs [26], and we hence termed them CD8 Tregs. Contrary to the overactivation seen in CD4 Tregs in aged mice, these CD8 Tregs displayed a shift from an immunosuppressive state to a cytotoxic state. This plasticity may provide foundational insights into the functional regulation of this particular cell subset. Interestingly, while humans do not possess the Klra gene found in mice, killer immunoglobulin-like receptors (KIR) are generally considered their human equivalent. Recent work by the Davis team has reported functional equivalence between KIR+CD8 T cells in humans and ly49+CD8+Tregs in mice, which we named CD8 Tregs in our study. Their research found that KIR+CD8 T cells can suppress inflammatory responses, thereby mitigating the damage caused by autoimmune diseases [67]. This undoubtedly further elevates the translational value of CD8 Tregs. In our study, we found a significant reduction in the number of CD8 Tregs both in the peripheral blood and ischemic brain of aged mice compared to young mice. Whether this trend will be confirmed in elderly human populations, and its implications for immune aging, are also of significant value.

In addition to the discovery of regulatory T cells, we observed an increased proportion of effector CD4 T cells such as Th1 and Th17. Previous studies have already reported an increase in the number of Th17 cells in the peripheral blood of elderly humans and mice [68], and our study aligns with these findings. We found that Th17 cells also extensively infiltrated the brain, with a notably higher proportion in aged mice compared to young counterparts. Furthermore, it is worth mentioning that Th1 cells were notably elevated in the peripheral blood of aged mice. These classic immune cells highly express interferon genes and could be one of the primary sources of interferon secretion in the aging process. A recent study found that the activation of the cyclic GMP–AMP synthase–stimulator of interferon genes signaling pathway is an essential contributor to the age-related type I IFN response in brain microglia [69]. This suggests that intervening in this pathway to regulate Th17 and IFN-related functions may help alleviate immunosenescence.

For CD8 T cells, besides the notable increase in the number of classical effector function cells like CD8 EF and CD8 EM, what is particularly attention-grabbing is the significant increase in CD8 EXL cells. Here, we found that although CD8 EXL cells in aged mice express PD-1 and other inhibitory immune receptors, they still maintain strong cytotoxicity and cytokine secretion capabilities. Previous studies have also reported similar findings [29,36]. Furthermore, upon entering the brain, the proinflammatory functions of CD8 EXL were further enhanced.

These observations may suggest that the CD8 exhaustion we have noted is part of a dynamic, progressive process. CD8 EXL cells are not terminally differentiated CD8 cells but are instead cells that are in the process of becoming exhausted due to overactivation, hence they still retain strong functionalities. We observed characteristic expression of Tox in CD8 EXL. Previous research has reported that Tox can promote CD8 exhaustion [70–73]. Importantly, it may be involved in the entire process where CD8 transitions from being overactivated to becoming exhausted. These observations suggest that intervening in Tox expression might help to inhibit CD8 exhaustion in the elderly, thereby reducing the emergence of highly proinflammatory CD8 T cell subgroups during this process.

There are several limitations to our study. Firstly, our current study did not undertake a comprehensive examination of peripheral immune cells across various age stages, from young to old. This leaves a gap in understanding age-related patterns, which could otherwise help in developing targeted interventions at specific time points. Future research may aim to address this shortfall. Additionally, using cell-tracking technologies to examine how aging peripheral immune cells behave when infiltrating the ischemic brain could offer further valuable insights. Lastly, although our dataset does include changes in resident brain immune cells, microglia, after ischemic stroke during aging, the current study primarily focuses on peripheral immune cells and thus may not fully capture the role of these resident cells.

In summary, our current study unveils the characteristics of peripheral blood immunosenescence in aged mice. These characteristics include accelerated neutrophil senescence, elevated antigen-presentation functions in MM, and an imbalance in T cell immunity. Importantly, these age-related changes in the peripheral immune system are not only present but also appear to be amplified when these cells infiltrate into the ischemic brain regions following an ischemic stroke.

## Materials and Methods

### Animals

Young (8 to 10 wk old) male C57BL/6 mice were purchased from Shanghai SLAC Laboratory Co. Ltd (Shanghai, China). Aged (20 to 22 mo old) male C57BL/6 mice were obtained from Beijing Vital River Laboratory Animal Technology Co. Ltd. (Beijing, China). All animals utilized for in vivo experiments were quartered in suitable temperature and humidity-controlled plastic cages with a 12-h light/dark period, where plentiful water and food were provided. Every endeavor was made to minimize animal suffering and the quantity of mice used.

All animal procedures were authorized by the Institutional Ethics Committee of the Second Affiliated Hospital, School of Medicine, Zhejiang University, which were also consent to National Institutes of Health Guide for the Care and Use of Laboratory Animals.

### Murine models of transient middle cerebral artery occlusion

Transient middle cerebral artery occlusion (MCAO) model was induced based on previous literature as described [74,75]. Briefly, mice were deeply anesthetized with 1% pentobarbital and then secured onto a plastic board while they were unresponsive to tail pinch tests. After carefully separating the common carotid artery (CCA), external carotid artery (ECA) and internal carotid artery (ICA), a silicone-coated filament with its head was inserted into ECA via a small incision. Subsequently, the filament was adjusted direction and advanced into ICA until resistance was perceived, thus, the original of the middle cerebral artery (MCA) was blocked and transient cerebral ischemia was induced. The filament was left at that position for 60 min and withdrawn to restore cerebral blood flow then, after which, the residual segment of ECA was knotted and the skin of the neck was sutured cautiously. 30% O_2_ /70% N_2_O was provided through nasal cannula to guarantee normal respiration, and the body temperature was maintained at 37.0 ± 0.5 °C during entire operation processes. Sham group mice underwent same operation without occlusion of MCA. The mice were placed on a warming blanket after surgery until they regained consciousness from anesthesia. The surgical personals were unaware of the grouping of mice.

### Behavior tests

We employed the rotarod test and adhesive test, which that have been previously reported to have high accuracy and sensitivity in estimating neurological deficits in rodents post stroke [76–78]. Behavior tests performers were blinded to the surgeon who established MCAO models, as well as the grouping of the mice. All behavior tests data were presented as the average of 3 duplicated trials per day, and a minimum interval between each trial was 15 min.

#### Rotarod test

Summarily, mice were coerced to run on a rotating apparatus (Unibiolab Biotech Center, Beijing, China) with an initial speed at 5 rpm and accelerating to 65 rpm within 300 s. The time consumed when the mice fell off the machine was recorded as latency to fall to assess locomotor function.

#### Adhesive test

In short, 2 × 3 mm stickers were attached to the forelimb contralateral to ischemic hemisphere. The time taken to recognize and take away the stickers was recorded to assess the post-stroke sensory function and motor ability of the mice individually. The maximum time for perception is 60 s and for stickers clearance is 120 s.

### Measurement of tissue loss

Mice were deeply anesthetized, followed by irrigating with 25 ml of phosphate-buffered saline (PBS) and 10 ml of paraformaldehyde sequentially. Brains of mice were excised and immersed in 4% paraformaldehydefor 24 h to fix. Afterwards, brain samples were dehydrated with a gradient of 15% to 30% sucrose solution at 4 °C [79]. After sinking at bottom of centrifuge tubes, the brain samples were sectioned into 6 equally thick layers with cryo-microtome, in which each layer contains 15 to 20 slices with 25-μm thickness. Cryoprotectants solution (40% PBS, 30% glycerol, and 30% ethylene glycol) was utilized to store these brain sections at −20 °C until needed. For tissue loss measurement, 1 slice in each layer (totally 6 slices) was selected for microtube associated protein 2 (MAP2) antibody (Proteintech, 17490-1-AP, 1:200) staining. The sum of the volume of normal hemisphere subtracted by the volume of the noninfarcted area in the ischemic hemisphere in each layer was defined as the tissue loss of mice brain.

### Immunofluorescence staining

Briefly, brain slices per mouse was/were selected and rinsed with PBS twice for 5 min and 0.5% Triton-X in PBS (PBST) treatment for 15 min at room temperature was used to facilitate cell membrane permeabilization. Then the slices were washed 3 times with 0.3% PBST for 5 min each. Subsequently, 5% normal donkey serum (in 0.3% PBST) was applied for blocking the brain sections for 1 h at room temperature, after which, the brain sections were incubated with following primary antibodies overnight at 4 °C: anti-Ly6G (Proteintech, RB6-8C5, 1:50), anti-CD31 (R&D, AF3628, 1:200), anti-Occludin (Abcam, ab216327, 1:100), anti-IBA-1 (Abcam, ab5076, 1:200), anti-MHC-II (ThermoFisher, 14-5321-85, 1:100), anti-GPNMB (Abcam, ab188222, 1:200), anti-FOXP3 (ThermoFisher, 14-5773-82, 1:200), anti-CD4 (ThermoFisher, 14-0042-82, 1:100), anti-CD8a (Abcam, ab217344, 1:200), anti-PD1 (Abcam, ab214421, 1:200). On the next day, the brain sections were rinsed with 0.3% PBST 3 times for 10 min each, and then incubated with corresponding secondary antibodies which were conjugated with Alexa Fluor 488, 555, 594, or 647 (Invitrogen, 1:500) in dim space at room temperature for 1 h. After washing with PBS 3 times, the brain sections were finally mounted on glass slides with 4′,6-diamidino-2-phenylindole-containing mount G (Abcam) and covered with coverslips.

For measurements of endogenous IgG leakage, brain sections were blocked in 5% bovine serum albumin (BSA) for 1 h, followed by incubation with donkey anti-mouse IgG antibody (Invitrogen, 1:500).

Leica TCS SP8 laser scanning confocal microscope (Leica Microsystems) was utilized for acquiring immunofluorescent images of brain sections.

### Imaris 3-dimensional rendering

To enable spatial visualization, the immunofluorescence images of MHC^+^ or GPNMB^+^ macrophages were subjected to 3-dimensional reconstruction using Imaris software (Version 9.01; Bitplane, Zurich, Switzerland) as described previously [80]. In short, images stacks obtained from confocal microscope were imported into Imaris and “surface module*”* tool were utilized for realizing 3-dimensional reconstruction for each spectral channel: structure reconstruction was conducted with the absolute fluorescence intensity from each channel in the regions of interest, and smoothing was achieved with distinct channel settings, as well as threshold setting to discern target signals from background noise and also eliminate nonspecific signals.

### T cells isolation and adoptive transfer

Spleen, cervical, axillary, and inguinal lymph nodes were harvested from noninjured aged or young mice to extract T cells as described before [81]. Briefly, the aforementioned tissues were mechanically minced and subjected to density gradient centrifugation using Ficoll. Subsequently, T cells were isolated using a CD3ε Microbeads kit (Miltenyi, 130-094-973) according to the manufacturer’s instructions. For in vivo experiment, 2 × 10^6^ isolated T cells from aged or young mice was transfer intravenously to recipient young mice 6 h after MCAO through tail vein respectively.

### Peripheral immune cells isolation and adoptive transfer

Whole blood was drawn from the right atrium of aged or young mice. Red blood cell (RBC) lysis buffer (1×; eBioscience, 00-4333-57) was used to lyse RBCs and obtain peripheral immune cells. In detail, 5 ml of 1× RBC lysis was added into fresh collected blood and incubated for 5 min; then, 8 ml of PBS was added, mixed well, and centrifuged (500g, 5 min), and the supernatant was discarded. After repeating this process 3 times, the pellet was resuspended with 1 ml of precooled PBS to prepare single-cell suspension of peripheral immune cells. For in vivo experiment, 2 × 10^6^ isolated immune cells from aged or young mice were transferred intravenously to young recipient mice 6 h after MCAO through tail vein respectively.

### Flow cytometry

Preparation of single-cell suspension of intracranial immune cells was performed as described previously [41]. In brief, mice were deeply anesthetized and perfused transcardially with 20 ml of precooled PBS. The brain was then dissected and the ischemic hemisphere was collected. Neural Tissue Dissociation Kit(T) (Miltenyi, 130-093-231) was used to dissociated brain samples to homogenates with a gentleMACS Octo Dissociator with Heaters (Miltenyi Biotec) following the instructions provided in manual. After passing through a 70-μm cell strainer and centrifuging (400g, 10 min), the pellet was resuspended with 10 ml of 30% Percoll, followed by density gradient centrifugation using 30%/70% Percoll (800g, 30 min). Subsequently, the intermediate layer containing the desired cells was collected, washed with PBS, and incubated with the following antibodies to surface antigens in dim spaces for 30 min on ice:anti-CD45-FITC (BD, 553079), anti-CD45-Pacific blue (Biolegend, 103126), anti-CD11b-PE-cy7 (Biolegend, 101216), anti-Ly6G-PerCP-cy5.5 (Biolegend, 127617), anti-NK1.1-BV605 (Biolegend, 108740), anti-CD3e-FITC (BD, 553061), anti-CD3e-APC (Biolegend, 100311), anti-CD4-APC-CY7 (BD, 552051), anti-CD25-BV605 (BD, 563061), anti-FOXP3-PE (BD, 566881), anti-PD-1-BV421 (Biolegend, 109121), anti-CD8a-APC (BD, 553035), anti-CD122-PE (BD, 553362), anti-Ly49G2-BV605 (BD, 742881), and anti-Cd62L-BUV395 (BD, 740218). According to the manufacturer’s instructions, anti-TGF-β1 antibody (Biolegend, 141302) was labeled with FITC fluorescence using the FITC Antibody Labeling Kit (AAT Bioquest, 1299) for flow cytometry experiments to detect the expression of TGF-β1 in Tregs.

Fluorochrome compensation was conducted with single-stained UltraComp eBeads (Thermo Fisher Invitrogen). Flow cytometry was performed on the Beckman CytoFELXIX flow cytometer (Beckman). FlowJo software was used to analyze data.

### Mass cytometry staining, data acquisition

The whole blood cells extracted from young or aged mice were subjected to RBC lysis and collected into a single-cell suspension. The isolated cells underwent a single rinse with 1× PBS and were then marked with a 250 nM cisplatin solution (Fluidigm) for 5 min in a chilled environment to exclude nonviable cells. Subsequently, cells were pretreated with Fc receptor blockers before incubation with a mixed antibody cocktail for surface markers for half an hour at low temperature. After a double rinse with a fluorescence-activated cell sorting-compatible buffer solution (1× PBS containing 0.5% BSA), cells were secured in an intercalation medium (Maxpar Fix and Perm Buffer with 250 nM 191/193Ir, Fluidigm) for an overnight period. Post-fixation, cells underwent a single rinse with both fluorescence-activated cell sorting buffer and perm buffer (eBioscience), followed by a half-hour incubation with an intracellular antibody mixture in a chilled setting. Finally, cells were purified and resuspended in deionized water, combined with 20% EQ beads (Fluidigm), and analyzed using a mass cytometer (Helios, Fluidigm).

### CyTOF data analysis

The analysis initiates by isolating individual samples from the comprehensive raw data through a dual-filtering mechanism, leveraging unique mass-tagged identifiers [82]. Subsequently, the generated .fcs files from various experimental runs are standardized using a bead-based normalization technique [83]. Cellular debris, dead cells, and cellular aggregates were then eliminated by manual gating strategies, retaining only live, isolated immune cells for further analysis. The cells are categorized into distinct phenotypic groups based on their marker expression profiles using the Phenograph clustering methodology [84]. Cell type labels are assigned to these clusters based on a heatmap depicting the patterns of marker expression across them. To provide a graphical representation of the data, the t-distributed stochastic neighbor embedding dimensionality reduction technique is adopted, displaying the spatial distribution of clusters and variations in marker expression among different samples or groups in a 2-dimensional format. Finally, the frequencies of these annotated cell populations are statistically evaluated using *t* test-based assessments.

In our targeted subpopulation analyses, manual gating procedures were executed using FlowJo (10.6.2) for delineation of specific cellular subtypes. SPADE analyses were conducted using the Cytobank Premium platform (Cytobank Inc.).

### Collection of immune cells and scRNA-seq

To understand the impact of aging on peripheral immune cells and the immune microenvironment following MCAO, we isolated total blood from both young and aged mice and ischemic brain immune cells from aged MCAO mice. Single-cell sequencing data of young mice was carried out in our previous study [41] and uploaded to the National Institutes of Health Gene Expression Omnibus database (GSE171171). Flow cytometric sorting identified CD45hi brain immune cells at 14 d post-MCAO. Cells were washed twice with PBS and resuspended with PBS containing 0.04% BSA into single-cell suspension. Subsequent cell processing and data acquisition were performed by Novogene (Beijing, China). Briefly, cells were loaded on a Chromium Single-Cell Controller Instrument (10x Genomics). scRNA-seq libraries were obtained by using the 10x Genomics recommended protocol. Every library was sequenced on an Illumina NovaSeq with 2 × 150 paired-end kits at Novogene.

### Preliminary data analysis of scRNA-seq (quality control, normalization, dimension reduction, and clustering analysis)

We used FastQC to perform basic statistics on the quality of the raw reads. The Cell Ranger software pipeline (Version 7.0.0) [85] provided by 10x Genomics was used to analyze the FASTQ files. We compared the raw data with the mm10 reference mouse genome to generate the filtered gene-barcode matrix across samples, which contained cellular barcodes and gene counts. All analyses were performed using R software (version 4.2.1, Vienna, Austria) and RStudio. Graphs and numbers were mostly generated using the R packages ggplot2 (version 3.4.2) and ggpubr (version 0.6.0). The count matrixes were processed and integrated using the Seurat (Version 4.3.0) [86] and Harmony (Version 0.1.1) [87] R packages. To remove low-quality cells, we applied a criterion to filter out cells with either under 100 or more than 4,400 expressed genes and expressing more than 20% of mitochondrial genes. Then the filtered matrix was normalized in Seurat. We scaled data with top 2,000 most variable genes and used these variable genes for principal component analysis. We employed the FindNeighbors function in Seurat to identify nearest neighbors for graph-based clustering based on principal components. Subsequently, cell subtypes were delineated using the FindClusters function in Seurat. Cell populations were visualized using uniform manifold approximation and projection (UMAP).

### Differential-expression analysis and enrichment analysis

The FindAllMarkers and FindMarkers functions were used to identify differentially expressed genes (DEGs) between clusters. The nonparametric Wilcoxon rank-sum test was used to obtain *P* values for comparisons, and the adjusted *P* values, based on Bonferroni correction, for all genes in the dataset. We used Volcano to visualize DEGs based on gene expression after the log-transformed and scaling. The adjusted *P* value < 0.05 and |log2foldchange | > 0.25 was set as the threshold for significantly differential expression. Gene Ontology (GO) enrichment analysis of DEGs [88–90] were respectively performed and visualized using the clusterProfiler (version 4.0.5) and enrichplot (version 1.18.4) package [91,92] (with *P* < 0.05). We performed UCell (version 4.0.5) [93] to calculate enrichment scores of pathways, of which genes were downloaded from the AmiGO [94].

### Transcription factor regulon analysis

The analysis of the regulatory network and regulon activity was performed by pySCENIC [95]. The regulon activity (measured in area under the curve [AUC]) was analyzed by AUCell module of the pySCENIC, and the activity of these regulons is quantified via AUCell default threshold [96]. The differential-expression regulon was identified by Wilcoxon rank-sum test in “FindAllMarkers” function in R package Seurat. The scaled expression of regulon activity was used to generate a heatmap.

### Pseudotime trajectory analysis

To investigate the maturation for neutrophils, we utilized the R package Monocle (version 2.26.0) and Monocle3 (version 1.3.1) [97]. The cellular trajectory analysis was performed using the learn_graph function. The results of Pseudo-time analysis were visualized on the UMAP dimensional scatter plot. Pseudotime represents the evolutionary trajectory of the cells.

### Scoring of biological processes

We utilized the AddModuleScore function for biological processes scoring. The functional gene sets were collected based on GO terms and prior research (Table S3).

In line with previous literature, the neutrophil aging score was calculated by considering changes in gene expression associated with neutrophil senescence [14,19] (Table S4). It was determined by taking the weighted mean of the normalized expressions of the above genes, with weights set to either +1 or −1 based on their correlation with the aging process.

### Statistical analysis

Data are represented as the average plus or minus the SD. For the scRNA-seq datasets, we employed the Wilcoxon rank sum test for statistical scrutiny. For comparing the averages between 2 groups that demonstrated equal variances and followed normal distribution, we utilized the Student *t* test. When examining variances in averages across several groups, 1-way analysis of variance (ANOVA) was the method of choice. In instances where measurements were repeated over time across different groups, 2-way repeated-measures ANOVA was used. All these statistical procedures were carried out using either R (version 4.2.1) or GraphPad Prism (version 9.5.0), and a *P* value less than 0.05 was considered indicative of statistical significance.

## Data Availability

The data that support the findings of this study are available from the corresponding author upon reasonable request.
