# Supplementary material for: Age-Related Alterations in Peripheral Immune Landscape with Magnified Impact on Post-Stroke Brain
Source: Research (Wash D C). 2023 Dec 11;6:0287. doi: 10.34133/research.0287 (PMC10712880; doi:10.34133/research.0287)
Supplement: Supplementary 1 — Figs. S1 to S6 Tables S1 to S4 [file research.0287.f1.zip › Supplementary Figures.docx]

**
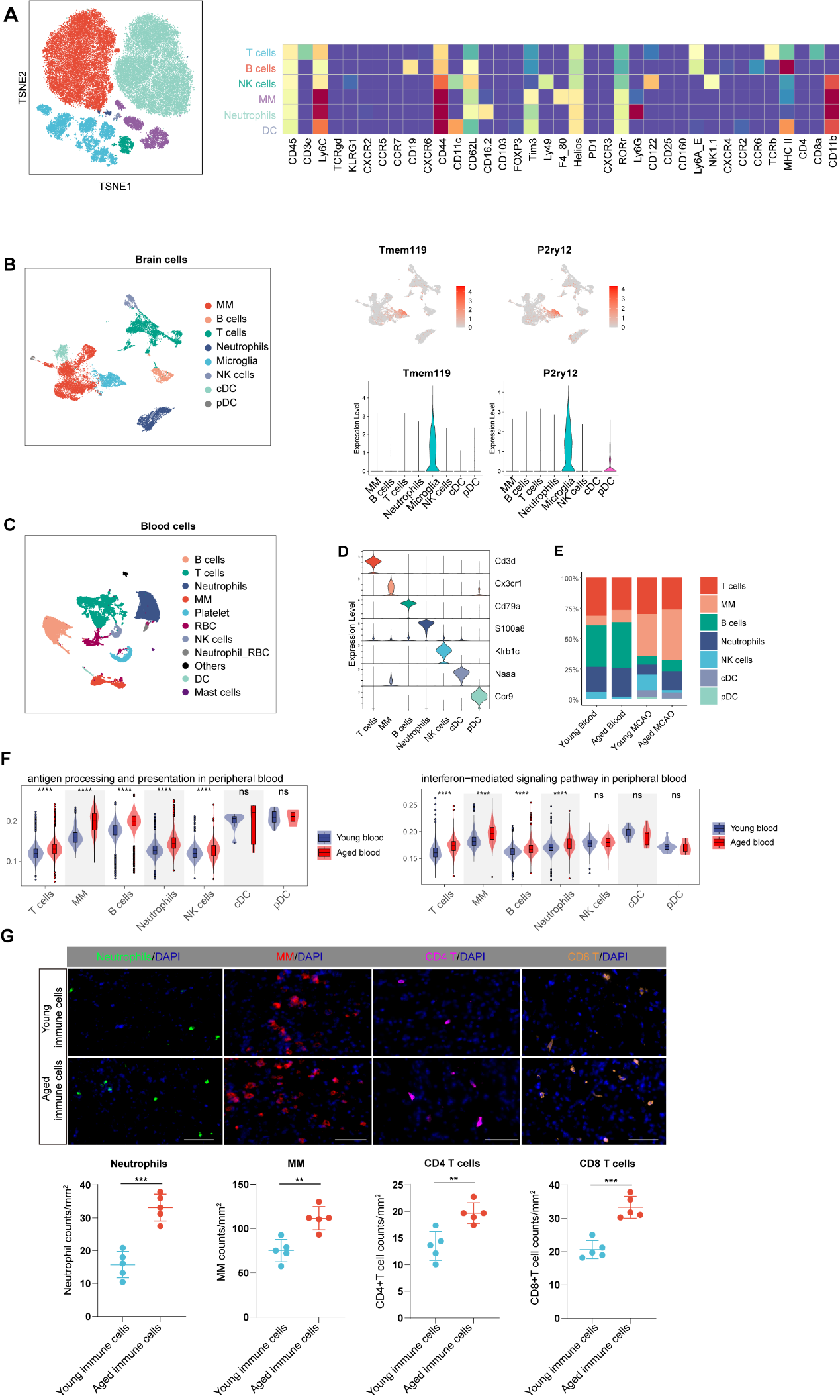
**

**Supplementary Figure 1.**

**A)** t-SNE dimensionality reduction of peripheral immune cells from aged and young mice, as detected by CyTOF (left). Heatmap showing the various markers of cell populations (right). **B-C)** UMAP projection plots displaying cell clusters of immune cells in the ischemic brain (B, left) and in the peripheral blood (C) of aged and young mice. Feature plots and violin plots illustrating the expression of microglial marker genes Tmem119 and P2ry12 (B, right). **D-E)** Violin plot D) showing the selected marker genes for each immune cell cluster in the blood and ischemic brain of both aged and young mice and stacked bar (E) plot illustrating the proportions of various immune cell populations. Both D) and E) are corresponding to Fig. 1C; **F)** Antigen-presenting functions and interferon pathways are significantly upregulated in various immune cell populations in the peripheral blood of aged mice compared to young mice. ****p ≤ 0.0001, Bonferroni-corrected Wilcoxon rank sum test; ns means not significant. **G)** Immunostaining of neutrophils, MM, CD4 T cells, and CD8 T cells suggest increased immune cell infiltration in the ischemic brain of mice receiving aged immune cells. n=5 per group. Scale bar = 50 um, **p<0.01, ***p<0.001, Student’s t-test.

**MM**: Monocytes/macrophages; **DC**: Dendritic cells

**
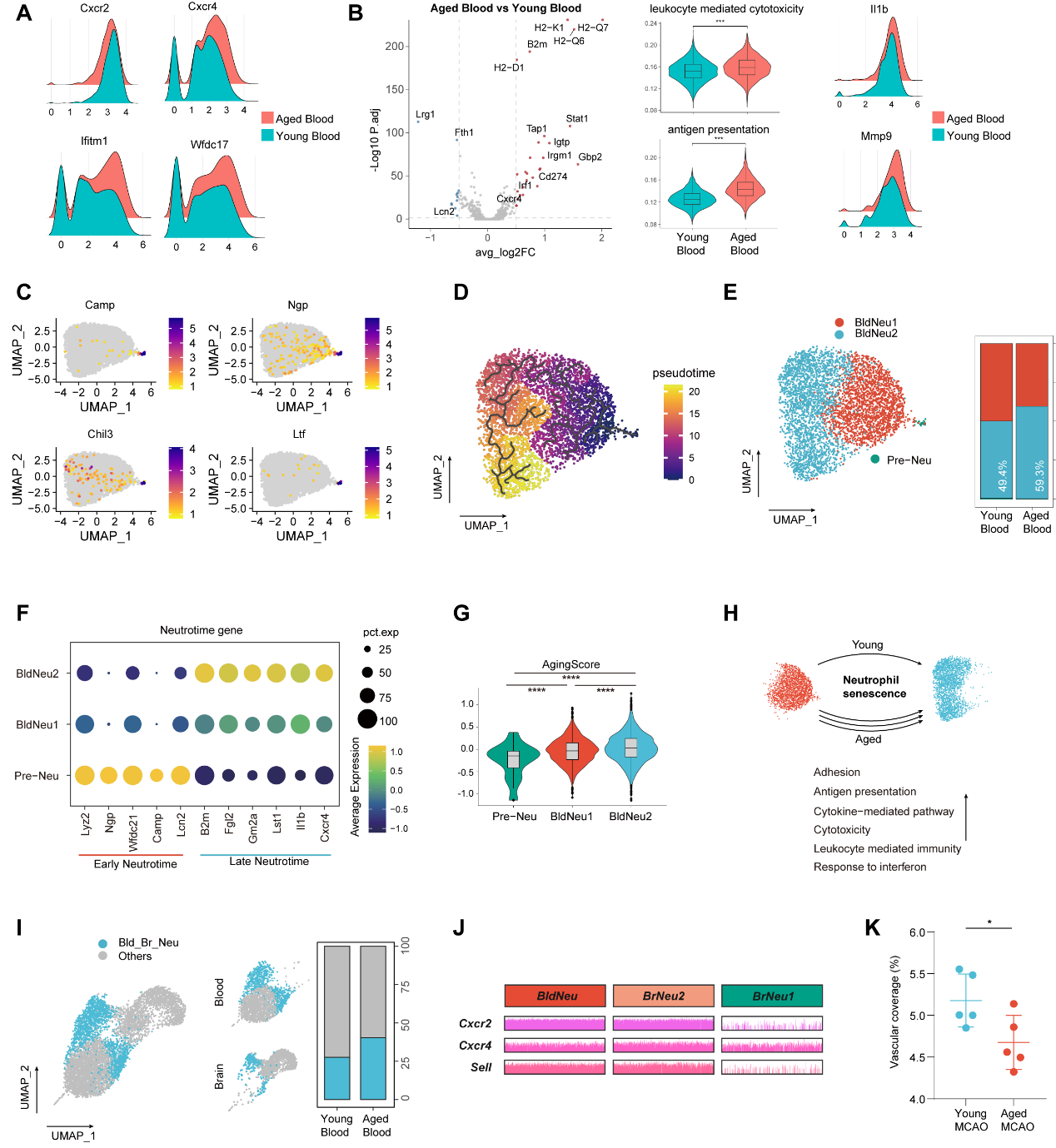
**

**Supplementary Figure 2.**

**A)** Ridge plot indicating downregulated expression of Cxcr2 and upregulated expression of Cxcr4, Ifitm1 and Wfdc17 in neutrophils of aged mice. Bonferroni-corrected Wilcoxon rank sum test. **B)** Differentially expressed genes between peripheral neutrophils in aged and young mice (left); upregulation of cytotoxicity and antigen-presentation functions in aged mice compared to young mice (mid); Ridge plot displaying elevated expression of Il1b and Mmp9 in peripheral neutrophils of aged mice (right). ***p<0.001, **C)** Feature plot illustrating high expression of immature neutrophil genes such as Camp, Ngp, Chil3, and Ltf in Pre-Neu cells. **D)** Pseudotime trajectory of peripheral neutrophils analyzed with Monocle3. **E)** UMAP plot of peripheral neutrophils from aged and young mice (left), and a stacked bar plot showing the proportion of the three clusters in each group (right). **F)** Expression of neutrotime-related genes in each cluster. **G)** Violin plot showing that BldNeu2 has the highest aging score. **** p ≤ 0.0001, Bonferroni-corrected Wilcoxon rank sum test. **H)** Accelerated neutrophil aging in aged mice compared to young mice, accompanied by improved immune function with the aging of neutrophils. **I)** Left: UMAP distribution of neutrophils that are mapped in both peripheral blood and ischemic brain (Bld_Br_Neu). Right: stacked bar plot showing the proportion of the Bld_Br_Neu in each group. **J)** Expression of aging-associated genes (Cxcr2, Cxcr4, and Sell) in BldNeu, BrNeu1, and BrNeu2. **K)** Aged mice exhibited lower vascular coverage at day 14 post-MCAO compared to young mice. n = 5 per group. *p<0.05, Student’s t-test.

**Pre-Neu**:Neutrophil precursor; **BldNeu**: Peripheral blood neutrophils: **BrNeu**: Neutrophil in the ischemic brain; **Bld_Br_Neu:** Neutrophils that are mapped in both peripheral blood and ischemic brain


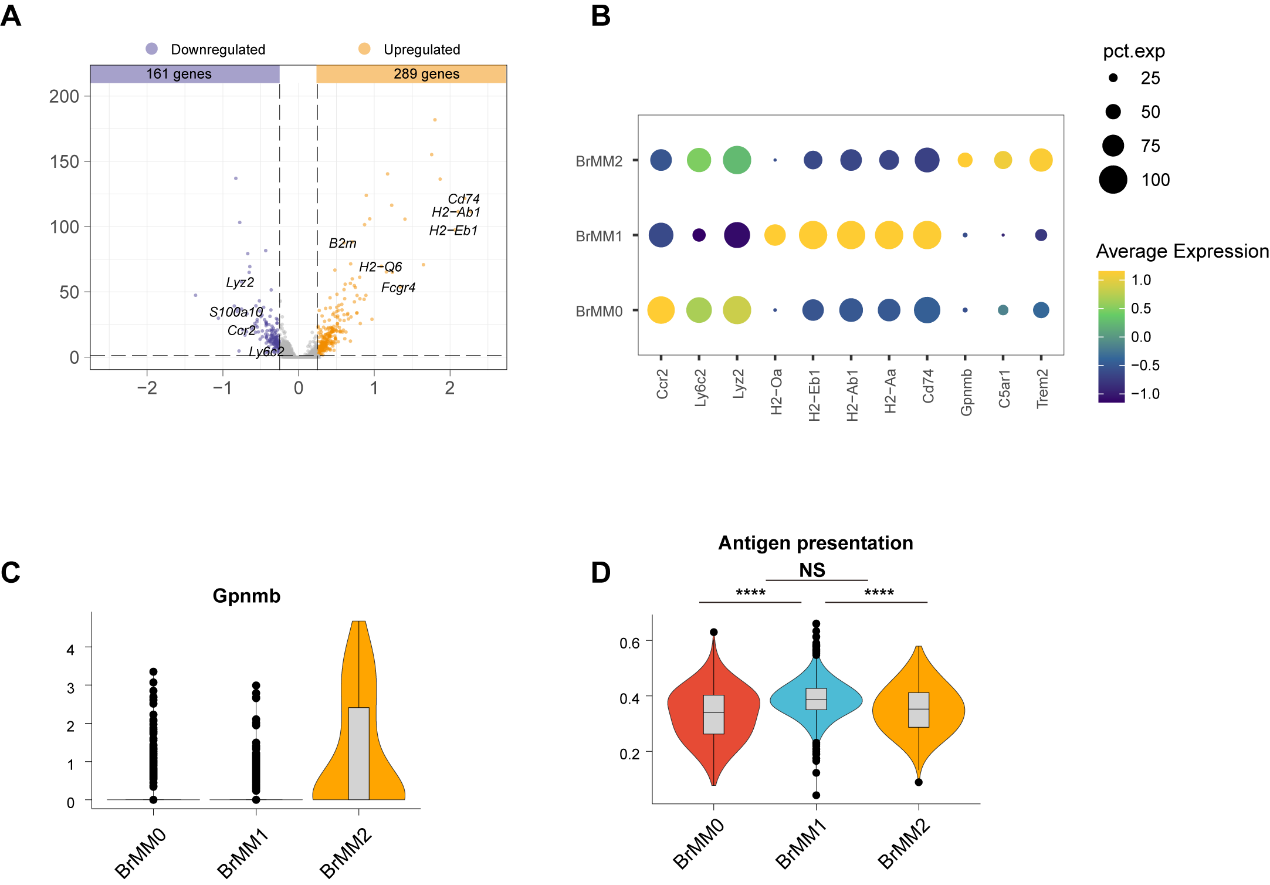


**Supplementary Figure 3.**

**A)** Volcano plot highlighting 161 down-regulated (in purple) and 289 up-regulated (in orange) differentially expressed genes (DEGs) with |log2 FC| > 0.25 and adjusted P < 0.05 in peripheral MM of aged mice compared to young mice. **B)** Dot plot illustrating key marker genes for the three MM clusters within the ischemic brain. **C-D)** Violin plots displaying heightened expression of the Gpnmb gene expression (C) in BrMM2 and elevated antigen-presentation functions (D) in BrMM1. **** p ≤ 0.0001, Bonferroni-corrected Wilcoxon rank sum test; NS means not significant.

**BldMM**: Peripheral blood monocytes/macrophages; **BrMM**: Monocytes/macrophages in the ischemic brain


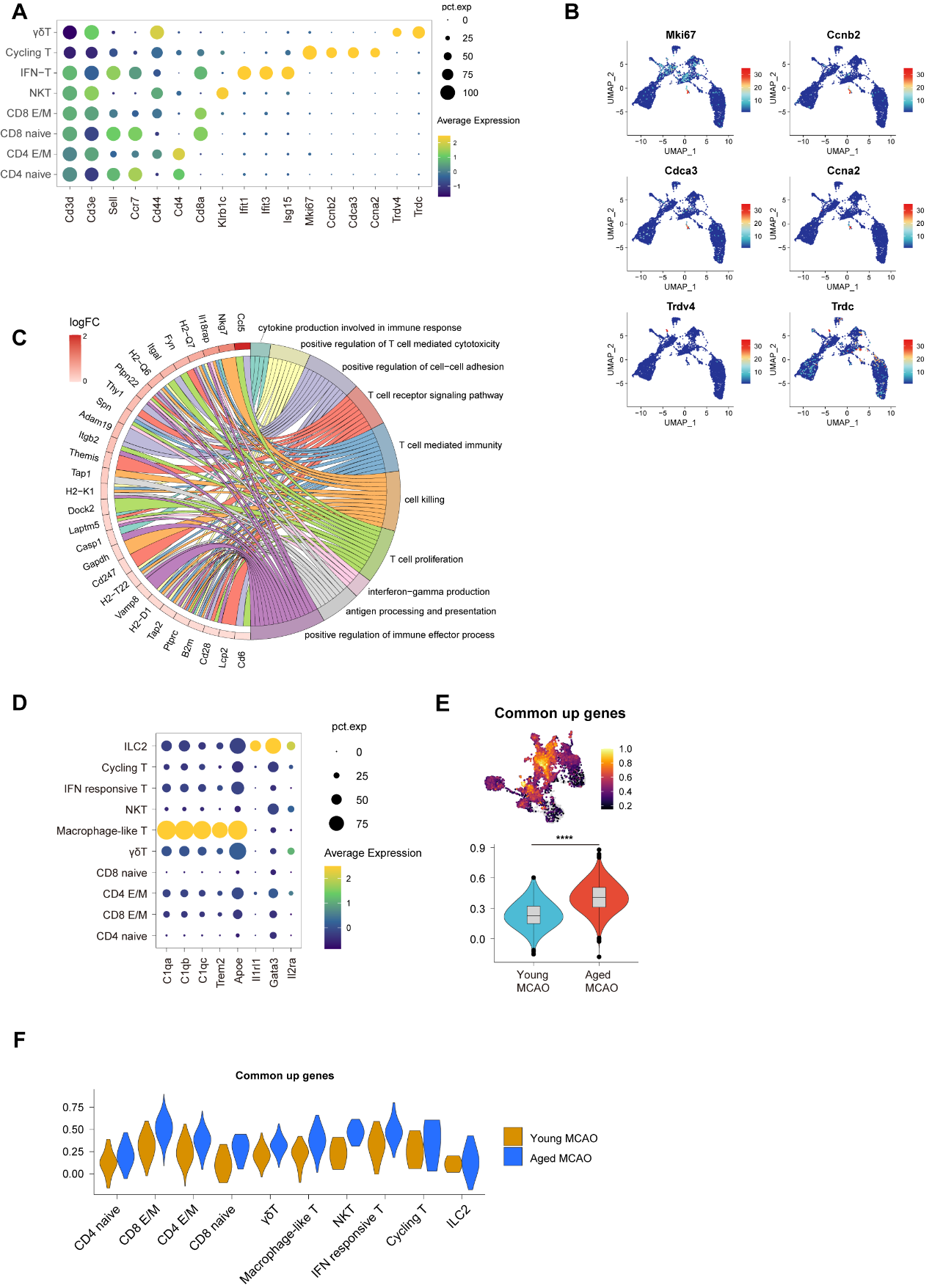


**Supplementary Figure 4.**

**A)** Dot plot illustrating marker genes for various T cell subpopulations in peripheral blood. B) Feature plot showing the expression of Mki67, Ccnb2, Cdca3, Ccna2, Trdv4 and Trdc in peripheral blood T cells. **C)** Chord diagram depicting functional enrichment of common up genes. **D)** Dot plot of marker genes for macrophage-like T and ILC2. **E)** Distribution of common up genes in CD4 E/M and CD8 E/M in the integrated T cell populations (top); these common up genes have higher expression levels in T cells from the aged ischemic brain compared to young (bottom). **** p ≤ 0.0001, Bonferroni-corrected Wilcoxon rank sum test. **F)** Expression differences of common up genes between aged and young T cell subpopulations in the ischemic brain.

**CD4 E/M**: CD4 effector/memory; **CD8 E/M**: CD8 effector/memory; **IFN-T**: T cells that respond to interferons

**
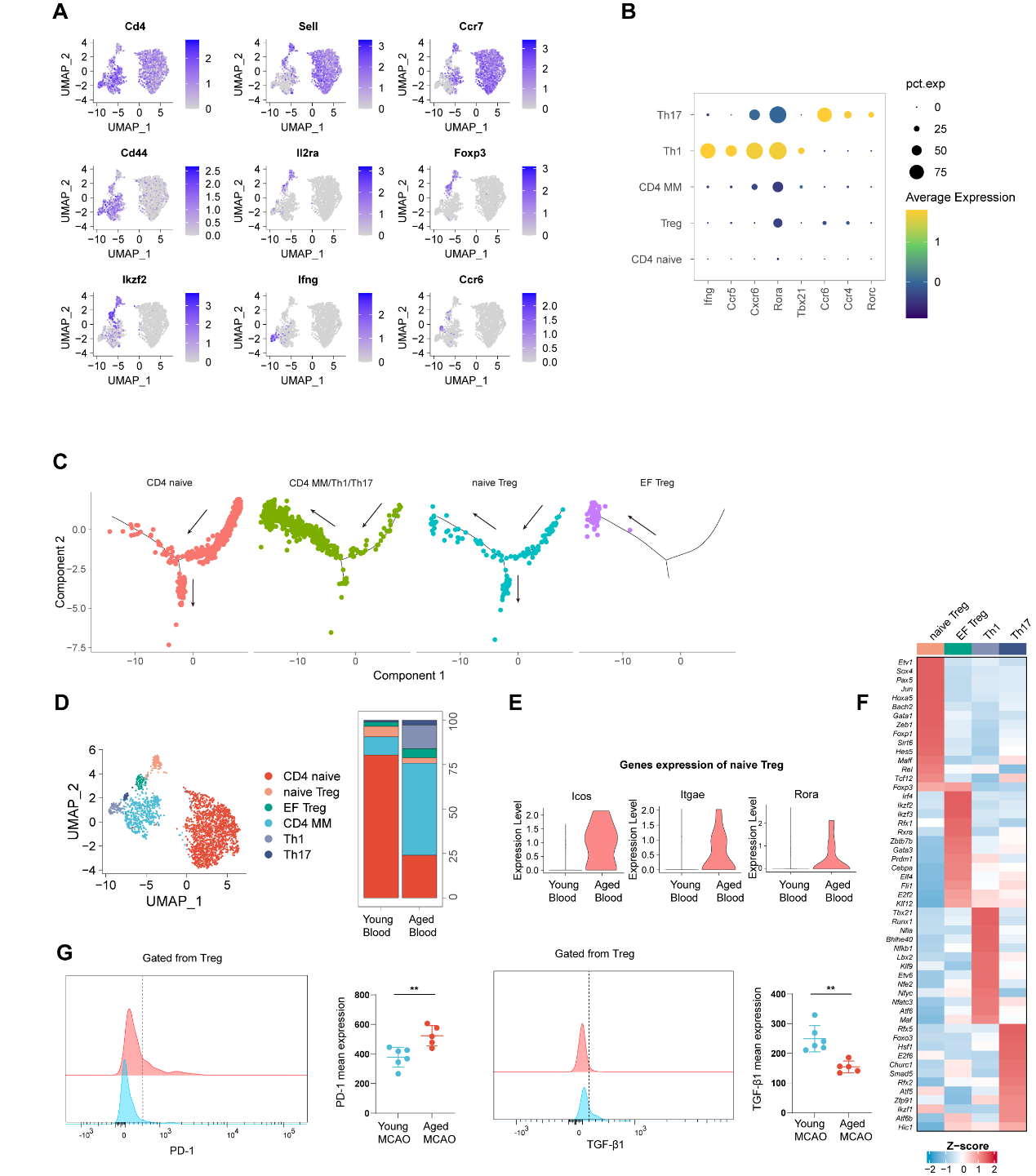
**

**Supplementary Figure 5.**

**A)** Feature plot showing marker genes for CD4 T cell subpopulations. **B)** Dot plot indicating marker genes for Th1 and Th17. **C)** Monocle pseudotime trajectory of CD4 T cell clusters. **D)** UMAP visualization of peripheral blood CD4 T cells, categorizing Tregs into naive Treg and EF Treg subsets (left), along with their respective proportions within the CD4 T cell pool (right). **E)** Violin plot indicating that peripheral naive Tregs in aged mice express higher levels of Icos, Itgae, and Rora compared to young mice. **F)** Heatmap showing the average regulon activities of representative transcription factors in CD4 T cell subset, derived from pySCENIC. The color gradient from blue to red indicates the relative expression levels from low to high. **G)** Flow cytometry analysis of PD-1 and TGF-β1 expression of Treg in the ischemic brain of aged mice (n=5) and young mice (n=6). **p<0.01, Student’s t-test.

**CD4MM**: CD4 memory; **EF Treg**: Effector Treg


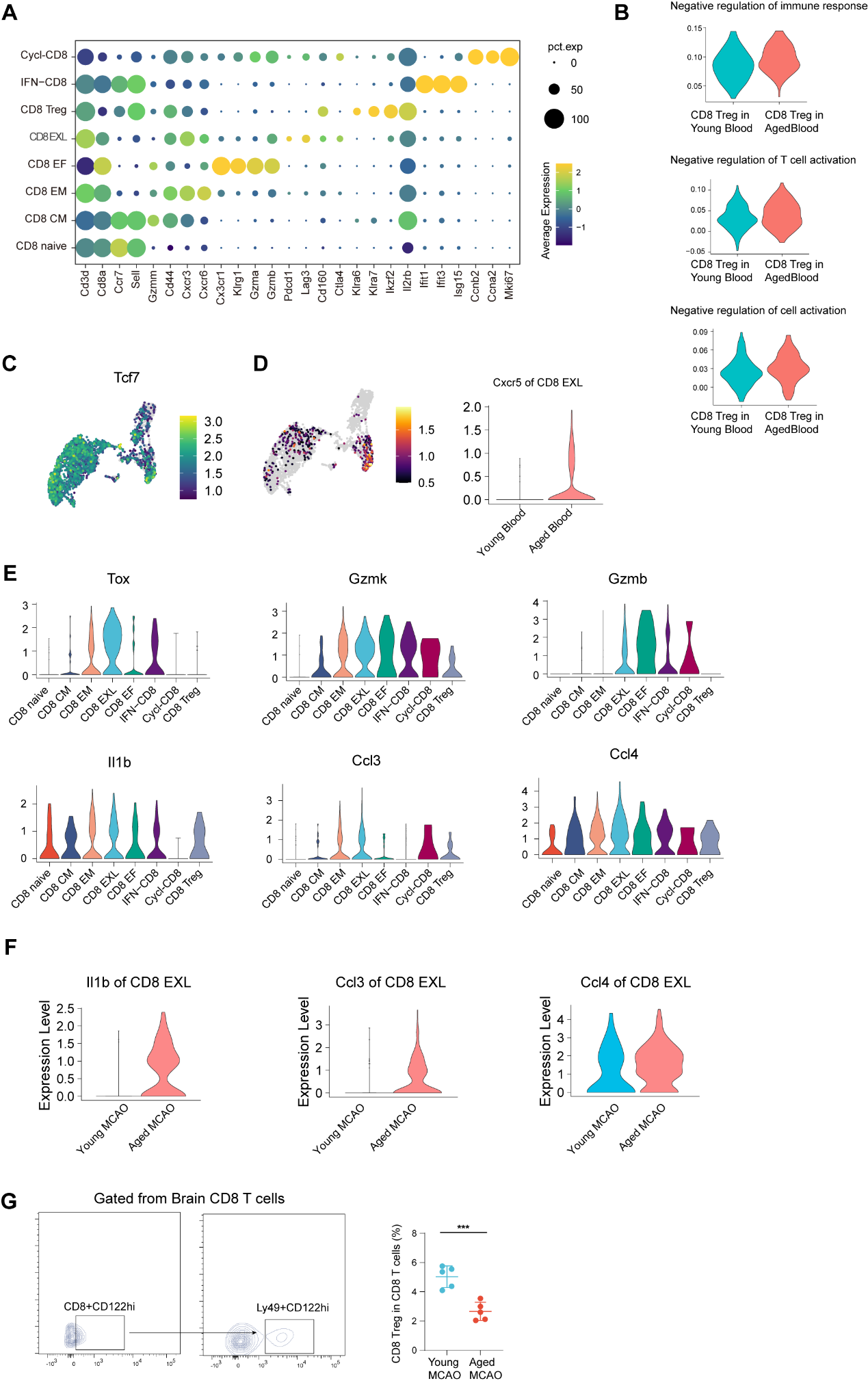


**Supplementary Figure 6.**

**A)** Dot plot illustrating marker genes for various CD8 T cell subpopulations. **B)** Violin plot demonstrating that there is no significant difference in immunoregulatory function between CD8 Treg cells in aged and young mice peripheral blood. **C)** Feature plot indicating Tcf7 expression in CD8 EXL. **D)** Feature plot of Cxcr5 gene expression (upper); Violin plot revealing higher Cxcr5 expression in aged CD8 EXL compared to young CD8 EXL. **E-F)** Violin plots showing the expression of Tox, Gzmk, Gzmb, Il1b, Ccl3 and Ccl4 of CD8 subpopulations in the ischemic brain (E), Il1b, Ccl3, and Ccl4 were expressed at higher levels in aged ischemic brains compared to young counterparts (F). **G)** Flow cytometry analysis of CD8 Treg cells in the ischemic brain of aged and young mice. n=5 per group. ***p<0.001, Student’s t-test.

**CD8 CM**: CD8 central memory; **CD8 EM**: CD8 effector memory; **CD8 EF**: CD8 effector; **CD8 EXL**: Exhausted-like CD8; **CD8 Treg**: Regulatory CD8 cells; **IFN-CD8**: CD8 T cells that respond to interferons; **Cycl-CD8**: Cycling CD8 T cells.
