# Supplementary material for: Age-Related Alterations in Peripheral Immune Landscape with Magnified Impact on Post-Stroke Brain
Source: Research (Wash D C). 2023 Dec 11;6:0287. doi: 10.34133/research.0287 (PMC10712880; doi:10.34133/research.0287)
Supplement: Supplementary 1 — Figs. S1 to S6 Tables S1 to S4 [file research.0287.f1.zip › Supplementary Table.pdf]

		MM				
**GO_0019882_antigen.processing.and.presentation**	**interferon pathway**	**GO_0002399_MHC.class.II.protein.complex.assembly**	**GO_0002449_lymphocyte.mediated.immunity**	**GO_1903037_regulation.of.leukocyte.cell.cell.adhesion**	**GO_0001910_regulation.of.leukocyte.mediated.cytotoxicity**	**GO_0034341_response.to.type.II.interferon**
Bag6	Ifnar2	H2-Aa	Ighv1-84	Ccl19	Klrc3	Ccl19
Mfsd6	Ifnar1	H2-Ob	Shld2	Dusp3	Cxcl1	Cdc42ep2
Washc1	Ifngr1	H2-Oa	Coro1a	Pde5a	Sh2d1b1	Ifitm6
Fcgr4	Ifngr2	H2-DMb2	Pou2f2	Laptm5	Nectin2	Ccl8
Pikfyve	Ifnz	H2-DMb1	Msh2	Coro1a	Ptprc	Sirpa
Slc11a1	Gm13283	H2-DMa	C1qbp	Cd274	Ripk3	Il23r
Psap	Ifng	H2-Eb2	Ighv1-9	Sirpa	Pik3r6	Ciita
Cd74	Dnaja3	H2-Eb1	Exo1	Tnfsf11	Cd160	Shfl
Traf6	Klhl20	H2-Ab1	Sh2d1b1	Dhps	F2rl1	Slc11a1
Ighm	Myd88	B2m	Il4ra	Cd80	Sh2d1a	Slc22a21
Ide	Tlr4	Iglc4	Ighv10-3	Cd86	Pomc	Tyk2
Ifi30	Ticam1		Foxp3	Il4ra	Il7r	Rab11fip5
Rab34	Hspd1		Fcgr4	Ripk2	Il12b	Gbp4
Icam1	Irf7		Myd88	Rara	Il12a	Jak1
Rab10	Irf3		Lig4	Foxp3	Igf2	Evl
Rab5b	Tlr3		Slc11a1	Rag1	Rasgrp1	Ccl9
H2-D1	Tbk1		Cebpg	Ptprc	Dnase1l3	Actg1
H2-Aa	Traf3ip1		Cd55	Pik3r6	Klre1	Syncrip
Pdia3	Ppme1		Cd55b	Wnt10b	Il23a	Stxbp4
Relb	Ap3d1		Mad2l2	Lrrc32	Il21	Tnf
Fcgr3	Ifih1		Mlh1	Scrib	Klrb1	Ifitm2
Wdfy4	Irf9		Aicda	Cd160	Lamp1	Stx8
Gm11127	Atg9a		Sh2d1a	Tnfrsf13c	Gimap3	Casp1
H2-T3	Nlrx1		Clec4g	Tyk2	Itgam	Il12b
H2-T23	Ap3b1		Il9r	Prkar1a	Gimap5	Cd74
H2-T22	Gadd45g		Il7r	Hes1	Stap1	Rpl13a
H2-Q7	Sh2d1b1		Il4	Cd55	Nckap1l	Tlr4
H2-Q6	Il18r1		Il2rb	Cd55b	Tyrobp	Daxx
H2-Q4	Ripk2		Il12b	Cd209b	H2-D1	Xcl1
H2-Q2	Foxp3		Il12a	Tnfrsf21	Crtam	Cxcl16
H2-Q10	Slc11a1		Cd74	Actb	Klri2	Il12rb1
H2-Ob	Itk		Ighm	Pla2g2e	Gm11127	Irf8
H2-Oa	Zg16		Ighd	Abl2	Ceacam1	Jak2
H2-DMb2	Abl1		Ighg1	Abl1	Dnase1	Cd47
H2-DMb1	Tnf		Ighg2b	Smarcd1	Klrd1	Gbp7
H2-DMa	Lta		Igha	Smarcd2	Klrk1	Vamp3
H2-M3	Il12b		Il18	Il1rl2	H2-T3	Capg
H2-M2	Il12a		Il21r	Zmiz1	H2-T23	Kif16b
H2-K1	Klre1		Il21	Card11	H2-T22	Gbp6
H2-Eb2	Il18		Rnf19b	Klf4	H2-Q7	Actr2
H2-Eb1	Il21		Icam1	Smarcb1	H2-Q6	Rab7b
Unc93b1	Rnf19b		Ighv1-54	Blm	H2-Q4	Dapk3
H2-Ab1	Irf8		Hspd1	Tnf	H2-Q2	Rps6kb1
Flt3	Il12rb2		Mpl	Cd83	H2-Q10	Gbp3
Ap3d1	Tcirg1		Nbn	Cdkn2a	H2-M3	Ccl12
Cd1d1	Sash3		Ighv3-8	Clec4g	H2-M2	Ccl22
Ifng	Nfkbiz		Jag1	Prnp	H2-K1	Myo18a
Rab32	Gata3		Cdh17	Il7r	Stat5a	H2-Aa
Ctse	Klrk1		Ighg3	Il7	Stat5b	Gsn
Ctss	Eomes		Ctsh	Il6st	Gfer	Acod1
Tapbp	Il20rb		Cd46	Il6	Pvr	Actr3
Rab6a	Il18rap		Csf3r	Il4	Cd1d1	Ifitm7
Ext1	Bcl3		Gapt	Il2rg	Cd226	Kynu
Kdm5d	Avpr2		Ighv1-53	Il2ra	Clec12b	Gch1
Ulbp1	Spn		Tcirg1	Il2	Arrb2	Gbp2b
Fcer2a	Nod2		H2-D1	Il1b	Serpinb9b	Gapdh
Rab35	Runx3		Fgl2	Il1a	Ulbp1	Mefv
Raet1e	Txk		Fcgr3	Il12b	Ppp3cb	H2-Q7
B2m	Azi2		Irf7	Il12a	Klrb1a	H2-Eb1

Treml4	Cd3e	Ighv9-4	Cd74	Klrb1b	Cx3cl1
Rftn1	Scgb1a1	Fas	Ihh	Klrb1c	H2-Ab1
Atg5	Enpp1	Ercc1	Kat5	Il18rap	Stat1
Raet1d	Shmt2	Batf	Traf6	Ap1g1	Ccl7
Psmb8	Ifitm6	Sash3	Dusp22	Raet1e	Igtp
Psmb9	Shfl	Nfkbiz	Igfbp2	B2m	Kif5b
Hfe	Tyk2	Gzmb	Igf2	Havcr2	Ifng
Rab33a	Jak1	Card9	Igf1	Arg1	Ifngr1
Mr1	Oas1g	Masp2	Ido1	Raet1d	Ifngr2
Rab27a	Ifitm2	Nkg7	Rasgrp1	Klri1	Aqp4
H60c	Hdac4	Klrd1	Hmgb1	Cxcl5	Stxbp2
H2-M5	Oas1e	H2-T23	Hlx	Hfe	Stxbp1
H2-Q1	Trim56	Ighv11-2	Il18	Klrc2	Stxbp3
H2-T24	Ifitm7	Ighv15-2	Zc3h12a	Klrc1	Stx11
Psme1	Setd2	H2-DMa	Zdhhc21	Lgals9	Rab12
Psme2	Stat1	H2-K1	Il23a	Mr1	Ifitm1
Rab8b	Oas1a	Unc93b1	Il21	P2rx7	Mrc1
Fcer1g	Oas2	H2-Ab1	Anxa1	H60c	Irgm1
Ap3b1	Ifitm1	Stat5a	Lgals3	H2-M5	Cdc42ep4
Rab3c	Sin3a	Stat5b	Lgals1	H2-Q1	Ccl17
Tapbpl	Ikbke	Stat6	Lef1	H2-T24	Slc22a5
Arl8b	Smpd1	Il31ra	Lck	Stx7	Ccl24
Mill2	Irak1	Ighv1-59	Gimap3	Fadd	Irf1
Calr	Ifitm3	Ighv9-2	Xcl1	Klrb1f	Gbp10
Tap2	Trim65	Icosl	Il12rb1	Clec2d	Snca
Tap1	Sp100	Csf2rb2	Socs1	Spi1	Ifitm3
Ctsl	Stat2	Traf3ip2	Tnfsf4	Ccl2	Ccl25
Iglc4	Isg15	Gzmm	Twsg1	Ager	Dnaja3
Rab3b	Oas1c	Iglc2	Icam1	Slamf6	Slk
Fcgr1	Ch25h	Iglc1	Rasal3	Vav1	Gbp9
Fcgr2b	Ccl19	Prdx1	Hsp90aa1	Cx3cr1	Vamp4
H60b	Cdc42ep2	Kdelr1	Hspd1	Cadm1	Rab20
Rab4a	Ccl8	Kif5b	Hspb1	Serpinb9	Gbp2
	Sirpa	Cd226	Jak2	Mill2	Rab43
	Il23r	Lyst	Cd47	Tap2	Zyx
	Ciita	Ighv1-64	Itgb2	Tap1	Pde12
	Slc22a21	Ighv1-22	Itgal	Crk	Sp100
	Rab11fip5	Ighv1-19	Itga4	Tgfb1	Tlr2
	Gbp4	Ighv7-3	Mad1l1	Lag3	Ccl6
	Evl	Ighv14-4	Ripor2	H60b	Ccl5
	Ccl9	Ighv1-50	Sirpb1a	Pnp	Ccl4
	Actg1	Ighv1-52	Cd209e		Ccl3
	Syncrip	Ighv1-80	Cd209d		Ccl2
	Stxbp4	Ighv1-76	Cd209c		Ccl1
	Stx8	Ighv1-78	Cd209a		Stx4a
	Casp1	Ighv1-75	Kitl		Flnb
	Cd74	Ighv1-55	Mdk		Cd40
	Rpl13a	Inpp5d	Ascl2		Vim
	Daxx	Iglc3	Pla2g2d		Trp53
	Xcl1	Ighg2c	Ccl28		Gbp8
	Cxcl16	Ighv1-61	Zfp608		Camk2a
	Il12rb1	Ighv2-3	Zbtb1		Calm1
	Jak2	Ighv9-1	Tarm1		Cdc42
	Cd47	Pirb	Gimap5		Slc26a6
	Gbp7	Bcl10	Smarcc1		Tgtp1
	Vamp3	C1ra	Arid1b		Myo1c
	Capg	Stx11	Tnfsf18		Trim21
	Kif16b	Serping1	Nckap1l		Vamp8
	Gbp6	Serpinb9b	Cd46		Gbp5
	Actr2	Ighv1-26	Selenok		Vps26b
	Rab7b	Ighv1-82	Pycard		Hpx
	Dapk3	Ighv1-34	Rps3		Was
	Rps6kb1	Ighv1-36	H2-D1		Dapk1
	Gbp3	Ighv1-81	H2-Aa		Bst2
	Ccl12	Ighv1-74	Gstp1		Nos2
	Ccl22	Ighv1-72	Btnl2		
	Myo18a	Ighv1-69	Rela		

H2-Aa		Ighv7-1	Fgl2
Gsn		Ighv10-1	Zbtb16
Acod1		Ighv6-6	Tmem131l
Actr3		Ighv14-2	Lax1
Kynu		Dlg1	Ets1
Gch1		Kdm5d	Zc3h12d
Gbp2b		Ighv1-58	Erbb2
Gapdh		Ighv5-16	Cyld
Mefv		Ighv4-1	Carmil2
H2-Q7		Ighv5-17	Pten
H2-Eb1		Ulbp1	Crtam
Cx3cl1		Ighv2-9-1	Cd6
H2-Ab1		Ighv2-6	Cxcl12
Ccl7		Ighv2-5	Ildr2
Igtp		Ighv5-12	Btla
Kif5b		Bcl6	Dock8
Aqp4		Ppp3cb	Sash3
Stxbp2		Ighv14-1	Nfkbiz
Stxbp1		Ighv5-4	Gm5150
Stxbp3		Ighv2-2	Lgals8
Stx11		Ighv5-2	Tnfrsf14
Rab12		Ighv1-47	Loxl3
Mrc1		Ighv1-39	Cblb
Irgm1		Ighv5-9	Ceacam1
Cdc42ep4		Ighv3-6	Ambra1
Ccl17		Ighv6-3	Gcnt1
Slc22a5		Ighv1-66	Gata3
Ccl24		Ighv5-6	H2-T3
Irf1		Ighv5-9-1	H2-T23
Gbp10		Il18rap	H2-T22
Snca		Aire	Vsir
Ccl25		Emp2	H2-Q7
Slk		Ptpn6	H2-Q6
Gbp9		Hc	H2-Q4
Vamp4		Atad5	H2-Q2
Rab20		Gzmc	H2-Q10
Gbp2		Fcer2a	H2-Ob
Rab43		Raet1e	H2-Oa
Zyx		Ebag9	H2-DMb2
Pde12		Bcl3	H2-DMb1
Tlr2		B2m	H2-DMa
Ccl6		Ighv8-12	H2-M2
Ccl5		Rnf8	Fcho1
Ccl4		Ighv13-2	H2-K1
Ccl3		Ighv14-3	H2-Eb2
Ccl2		Ighv1-18	H2-Eb1
Ccl1		Ighv1-15	Foxj1
Stx4a		Treml4	Nfat5
Flnb		Rftn1	H2-Ab1
Cd40		Ighv1-12	Stat5a
Vim		Raet1d	Stat5b
Trp53		Rnf168	Fut4
Gbp8		Shld1	Tespa1
Camk2a		Cd70	Mia3
Calm1		Shld3	Tnfsf14
Cdc42		Ighv1-85	Icosl
Slc26a6		Nod2	Zap70
Tgtp1		Ighv1-42	Smarca2
Myo1c		Ighv1-7	Arid1a
Trim21		Ighv1-5	Syk
Vamp8		P2rx7	Tnfsf13b
Gbp5		Myo1g	Casp3
Vps26b		C1rl	Ap3d1
Hpx		Rab27a	Smarcc2
Was		H60c	Ccdc88b
Dapk1		Exosc3	Ptpn2
Bst2		Fosl2	Klhl25

Nos2	Ctsc	Fut7
Traf3ip3	Ung	Cd1d1
Lamp3	Vamp7	Ifng
Ifi204	Cd81	Jak3
Tpr	Ephb6	Runx1
Axl	Ighv1-63	Cbfb
Gas6	Unc13d	Sirpb1b
Ifit1	Fcer1a	Zfp35
Ifit2	Fcer1g	Bcl10
Myc	Cfh	Dlg5
Star	Cd8a	Rc3h1
Eif2ak2	Cd40lg	Rhoh
Plscr1	Cd40	Cd276
Ro60	Stard7	Adora2a
Adar	Cd2	Il20rb
Ifi205	Cd19	Dlg1
Ifi207	Ighv9-3	Cd300a
Ifi209	Xrcc4	Bcl6
Ifi203	Ccr6	Ptpn22
Trim6	Serpinb9	Ppp3ca
Aim2	Slfn2	Malt1
Mavs	Msh6	Glmn
Gm5431	Csf2rb	Smad7
Plscr2	Crlf2	Irgm1
Irgm2	Arl8b	Socs5
Iigp1	C8g	Pag1
Ifi214	C4b	Ndfip1
Xaf1	C3	Phf10
Ifi206	C2	Itch
Gm4841	C1qc	Ptpn6
Htra2	C1qb	Skap1
Pnpt1	C1qa	Adam8
Ifi47	Btk	Tsc2
F830016B08Rik	Tap2	Itpkb
Mndal	Cd96	Zfp609
Ube2g2	Cr1l	Nrarp
9930111J21Rik1	Cr2	Arg2
Ifi211	Lag3	B2m
Ifi208	Swap70	Slc7a1
Ddx41	Ighv8-8	Ccr2
Ifit3	Fcgr1	Havcr2
Cdc34	Fcgr2b	Irf1
Ube2k	H60b	Spta1
Gm11772	Prkcd	Spn
Gm12185	Prf1	Sox4
Ndufa13	Tnfsf13	Sox13
Tgtp2	Plekhm2	Sox12
Ifi213	Enpp1	Ass1
		Arg1
		Ccr7
		Il15
		Irak1
		Ccl25
		Brd7
		Alox5
		Akt1
		Dnaja3
		Adk
		Ada
		Tigit
		Nfkbid
		Flot2
		Dusp10
		Icos
		Efnb3
		Hfe
		Cd59a

Gpam
Dpp4
Rc3h2
Tnfsf9
Lgals9
Zbtb7b
Prdx2
Cav1
Efnb1
Lilrb4a
Tnfaip8l2
Runx3
Slc4a1
Pbrm1
Dapl1
Wnk1
Actl6a
Nck1
H2-M5
H2-Q1
Il4i1
Zc3h8
Pdcd1lg2
Gli3
Cd59b
Tbx21
Slc4a2
Fadd
Cd81
Nck2
Slamf1
Ephb6
Bad
Cd244a
Shb
Selp
St3gal4
Sele
Ccl5
Ccl2
Ager
Aif1
Sart1
Cebpb
Cd5
Cd44
Cd40lg
Cd4
Cd3e
Cd37
Ptafr
Cd28
Cd27
Cd24a
Egr3
Xbp1
Arid2
Scgb1a1
Tfrc
Ap3b1
Nkap
Adtrp
Pawr
Gpnmb
Smarcd3
Gp1ba
Slfn1

Vcam1
Sirpb1c
Socs6
Peli1
Capn1
Smarca4
Bmi1
Ctsg
Ctla4
Iglc4
Thy1
Tgfbr2
Tgfb1
Lag3
Smarce1
Rhoa
Gnrh1
Dtx1
Nlrp3
Prkcz
Prkcq
Sdc4
Efnb2
Hsph1
Chst2
Nr4a3
Pnp
Tspan32

		Neutrophils					
**GO_0002697_regulation.of.immune.effector.process**		**GO_0001959_regulation.of.cytokine.mediated.signaling.pathway**	**GO_0031343_positive.regulation.of.cell.killing**	**GO_0034341_response.to.type.II.interferon**	**GO_0002705_positive.regulation.of.leukocyte.mediated.immunity**	**GO_0002831_regulation.of.response.to.biotic.stimulus**	**GO_1905603_regulation of blood-brain barrier permeability**
Klk7		Prkn	Klrc3	Ccl19	Nsd2	Gsdme	Sh3gl2
Ccl19		Laptm5	Cxcl1	Cdc42ep2	Klrc3	Klk7	Abcc8
Thoc1		Parp14	Sh2d1b1	Ifitm6	Cxcl1	Banf1	Tjp1
Nsd2		Traf2	Nectin2	Ccl8	Dhx36	Nop53	Angpt1
Klrc3		Angpt1	Ptprc	Sirpa	Shld2	Klrc3	Zeb2
Laptm5		Nlrp6	Cd160	Il23r	Fzd5	Cxcl1	Tjp2
Cxcl1		Ripk2	F2rl1	Ciita	Traf2	Parp14	Vegfa
Dhx36		Ptprc	Sh2d1a	Shfl	Msh2	Fbxl2	Tjp3
Il17f		Nkiras2	Pomc	Slc11a1	Cd177	Il17f	Ocln
Shld2		Rabgef1	Il12b	Slc22a21	Sh2d1b1	Slc15a3	
Fzd5		Mmp12	Il12a	Tyk2	Il18r1	Cd274	
Traf2		Ticam2	Rasgrp1	Rab11fip5	Foxp3	Usp15	
Msh2		Cpne1	Klre1	Gbp4	Nectin2	Ifi205	
Cd177		F2rl1	Il23a	Jak1	Ptprc	Ppm1b	
Cd80		Gps2	Il21	Evl	Cd160	Il23r	
Cd86		Gigyf2	Lamp1	Ccl9	Rif1	Cd86	
Angpt1		Trem2	Gimap3	Actg1	Cd55	Atg12	
C1qbp		Nkiras1	Itgam	Syncrip	Cd55b	Nfe2l2	
Sh2d1b1		Oas1g	Oga	Stxbp4	Mad2l2	C1qbp	
Il18r1		Med1	Gimap5	Tnf	Pms2	Smpdl3b	
Il4ra		Pparg	Stap1	Ifitm2	Ticam2	Nlrp6	
Rbp4		Il7	Tyrobp	Stx8	Mlh1	Ecsit	
Ripk2		Il6st	H2-D1	Casp1	F2rl1	Sh2d1b1	
Rara		Il6	Crtam	Il12b	Trem2	Hspa8	
Rap1a		Il1rn	Klri2	Cd74	Sh2d1a	Ifi207	
Foxp3		Il1r2	Gm11127	Rpl13a	Tnf	Lgr4	
Rac2		Il1r1	Klrd1	Tlr4	Lta	Ripk2	
Nectin2		Casp1	Klrk1	Daxx	Pomc	Foxp3	
Ptprc		Cd74	H2-T3	Xcl1	Il6	Nectin2	
Ripk3		Ythdf2	H2-T23	Cxcl16	Il4	Pik3r6	
Pik3r6		Nol3	H2-T22	Il12rb1	Il2	Ffar2	
Ffar2		Trim6	H2-Q7	Irf8	Il1r1	Traf3	
Pkn1		Ppp2cb	H2-Q6	Jak2	Il1b	Myd88	
Myd88		Rnf185	H2-Q4	Cd47	Il12b	Cd160	
Sirt1		Tnfrsf1a	H2-Q2	Gbp7	Il12a	Casp8	
Cd160		Hipk1	H2-Q10	Vamp3	Traf6	Rnf135	
Rabgef1		Mavs	H2-M3	Capg	Ighg1	Brcc3	
Rif1		Irgm2	H2-M2	Kif16b	Ighg2b	Mfhas1	
Cd55		Stap1	H2-K1	Gbp6	Clnk	Ubqln1	
Cd55b		Ripk1	Stat5a	Actr2	Rasgrp1	Cd55	
Adora3		Csf1	Stat5b	Rab7b	Klre1	Cd55b	
Mad2l2		Pycard	Syk	Dapk3	Il18	Gramd4	
Pms2		Irak3	Pvr	Rps6kb1	Il23a	Tyro3	
Snx4		Irf7	Cd1d1	Gbp3	Il21	Gbp4	
Ppl		Irf3	Ifng	Ccl12	Paxip1	Mmp12	
Ticam2		Oas1e	Cd226	Ccl22	Fbxo38	Ercc6	
Panx1		Cyld	Bcl2l11	Myo18a	Lamp1	Cd180	
Mlh1		Spata2	Cd5l	H2-Aa	Gimap3	Ppl	
F2rl1		Trim56	Ulbp1	Gsn	Xcl1	Ticam2	
Acp5		Pias4	Ccr5	Acod1	Tnfsf4	Tnip2	
Trem2		Tbk1	Klrb1c	Actr3	Ticam1	Nlrp1b	
Pld2		Trim44	Il18rap	Ifitm7	Hspd1	Rps6ka3	
Sh2d1a		Axl	Fcer2a	Kynu	Kmt5b	F2rl1	
Slamf8		Gfi1	Ap1g1	Gch1	Kit	Gps2	
Tnf		Gas6	Raet1e	Gbp2b	Itgb2	Gigyf2	
Lta		Usp27x	B2m	Gapdh	Itgam	Polr3b	
Prkdc		Ecm1	Arg1	Mefv	Scimp	Trem2	
Clec4g		Peli3	Raet1d	H2-Q7	Mavs	Tut4	
Susd4		Sigirr	Klri1	H2-Eb1	Zbtb1	Oas1g	

Pomc		Syk	Hfe	Cx3cl1	Gimap5	Unc13b
Il7r		Igtp	Klrc2	H2-Ab1	Stap1	Med1
Il6		Casp4	Klrc1	Stat1	Ddx1	Sh2d1a
Il4		Oas1a	Mr1	Ccl7	Tyrobp	Slamf8
Il2		Oas3	P2rx7	Igtp	H2-D1	Tnf
Il1r1		Ptpn2	H60c	Kif5b	Fcgr3	Prkdc
Casp1		Ttll12	H2-M5	Ifng	Crtam	Nfkbia
Il1b		Ythdf3	H2-Q1	Ifngr1	Klri2	Susd4
Il13		Mul1	H2-T24	Ifngr2	Sash3	Ifi209
Il12b		Irgm1	Stx7	Aqp4	Ddx21	Pparg
Il12a		Otud4	Fadd	Stxbp2	Gm11127	Pomc
Il10		Ikbke	Bad	Stxbp1	Gata3	Il4
Cd74		Padi2	Spi1	Stxbp3	Klrd1	Casp1
Traf6		Mmp8	Ccl2	Stx11	Klrk1	Il1b
Dusp22		Palm3	Slamf6	Rab12	H2-T3	Il12b
Trpm4		Arg1	Vav1	Ifitm1	H2-T23	Il12a
Ighm		Irak1	Cadm1	Mrc1	H2-T22	Cd74
Ighg1		Sharpin	Mill2	Irgm1	H2-Q7	Kat5
Ighg2b		Dnaja3	Tap2	Cdc42ep4	H2-Q6	Traf6
Igf2		Cactin	Lag3	Ccl17	H2-Q4	Igf2
Il27		Cav1	H60b	Slc22a5	H2-Q2	Ifi204
Ncf1		Ptprf	Prf1	Ccl24	H2-Q10	Ifi203
Clnk		Trim32	Pnp	Irf1	H2-M3	Tlr6
Rasgrp1		Rnf113a2	Nos2	Gbp10	H2-M2	Tlr1
Dnase1l3		Zbp1		Snca	H2-K1	Il27
Hmox1		Irak2		Ifitm3	Stat5a	Ncf1
Hmgb1		Txk		Ccl25	Stat5b	Clnk
Hlx		Traip		Dnaja3	Stat6	Ythdf2
Hk1		Samhd1		Slk	Dennd1b	Nr1d1
Klre1		Rnf113a1		Gbp9	6030468B19Rik	Nlrp10
Il18		Cxcr4		Vamp4	Pvr	Nlrc3
Zc3h12a		Vrk2		Rab20	Cd1d1	Rasgrp1
Trim6		Ube2k		Gbp2	Ifng	Hmgb2
Il23a		Cd300lf		Rab43	Cd226	Hexim1
Lyn		Fadd		Zyx	Kmt5c	Hmgb1
Il21		Mettl3		Pde12	Ulbp1	Klre1
Klrb1		Adam17		Sp100	Malt1	Pik3r1
Paxip1		Stat2		Tlr2	Klrb1c	Zc3h12a
Tlr4		Ccl5		Ccl6	Rsad2	Trim11
Anxa1		Sh2b3		Ccl5	Il18rap	Trim6
Tlr9		Robo1		Ccl4	Atad5	Il23a
Fbxo38		Tslp		Ccl3	Fcer2a	Lyn
Pdpk1		Isg15		Ccl2	Ap1g1	Il21
Lgals3		Cd24a		Ccl1	Raet1e	Klrb1
Slc15a4		Cnot7		Stx4a	B2m	Slc15a2
Lamp1		Naip2		Flnb	Itgb2l	Ltf
Gimap3		Naip1		Cd40	Arg1	Tlr4
Xcl1		Naip6		Vim	Raet1d	Ppp2ca
Rapgef1		Naip5		Trp53	Klri1	Tlr9
Pglyrp4		Oas1c		Gbp8	Shld1	Fbxo38
Ptprj		Otulin		Camk2a	Hfe	Pdpk1
Pla2g3		Rbm47		Calm1	Dpp4	Slc15a4
Tnfrsf4		Lsm14a		Cdc42	Shld3	Lamp2
Tnfsf4		Adipoq		Slc26a6	Klrc2	Lamp1
Tnfrsf1b		C1qtnf4		Tgtp1	Klrc1	Usp38
Rtn4		Adar		Myo1c	Nod2	Rnf185
Ticam1		Rffl		Trim21	Mr1	Trim3
Hspd1		Trim41		Vamp8	P2rx7	Pml
Kmt5b		Eif4e2		Gbp5	H60c	Gimap3
Kit		Edn1		Vps26b	Exosc3	Ccr1
Cd47		Hif1a		Hpx	H2-M5	Rab34
Itgb2		Nlrc5		Was	H2-Q1	Ly96
Itgam		Crebrf		Dapk1	H2-T24	Ly86
Ankrd17		Cdc37		Bst2	Stx7	Xcl1
Dnajb9		Pafah1b1		Nos2	Tbx21	Il12rb1
Nod1		Sphk1			Clcf1	Xrcc5
Mif		Hpx			Fadd	Nek7

Ascl2	Parp9	Cd81	Emilin2
Scimp		Slamf1	Rtn4
Mavs		Trp53bp1	Nploc4
Zbtb1		Spi1	Ticam1
Cgas		Ccl2	Hsp90aa1
Gimap5		Stx4a	Hspd1
Ube2j1		Fcer1a	Dtx4
Stap1		Fcer1g	Nlrc4
Tnfsf18		Cd40	Gbp7
Nckap1l		Ptafr	Naglu
Ddx1		Cd28	Ankrd17
Pram1		Cd24a	Htra1
Fer		Slamf6	Usp50
Cd46		Vav1	Nod1
Spon2		Tfrc	Mif
Gprc5b		Cadm1	Zmpste24
Tyrobp		Arid5a	Rab11fip2
Myo18a		Pagr1a	Scimp
Sema7a		Mill2	Aim2
Pycard		C3	Mavs
Irf5		Btk	Ppp2r3c
H2-D1		Tap2	Trem3
Grn		Tgfb1	Casp6
Irak3		Lag3	Tlr5
Prg2		Hmces	Rab7b
Fgl2		Map3k7	Cgas
Siglecg		Fcgr1	Pspc1
Fgr		H60b	Gimap5
Fes		Hpx	Atat1
Fcgr3		Nlrp3	Dtx3l
Tlr7		Plcg2	Plscr2
Tlr3		Prkcz	Tspan6
Pten		Tnfsf13	Gbp3
Crtam		Pnp	Irgm2
Ffar3			Tnip1
Klri2			Emilin1
Tnfaip3			Pcbp2
Sash3			Gpatch3
Pglyrp2			Zc3hav1
Nfkbiz			Fam3a
Phb2			Tyrobp
Ddx21			Clec4e
Card9			Serpinb1a
Gm11127			Zdhhc12
Tnfrsf14			S100a14
Loxl3			Cep63
Ceacam1			Selenok
Axl			Pycard
Dnase1			H2-D1
Gata3			Grn
Gata2			Irak3
Gata1			Syt11
Dhx58			Rela
Klrd1			Fgl2
Klrk1			Tkfc
H2-T3			Acod1
H2-T23			Fgr
H2-T22			Trim30d
Vsir			Irf7
H2-Q7			Trib1
H2-Q6			Mapkbp1
H2-Q4			Trim30c
H2-Q2			Trim30b
H2-Q10			Rnf125
H2-DMb2			Irf3
H2-DMb1			Oas1e
H2-M3			Tlr8

H2-M2	Tlr7
H2-K1	Tlr3
Foxj1	Pum2
Stat5a	Pum1
Stat5b	Pten
Stat6	Ifi214
Dennd1b	Crtam
6030468B19Rik	Klri2
Il27ra	Tnfaip3
Tmbim6	Sec14l1
Ephb2	Rnf26
Syk	Trim56
Appl2	Parp1
Abr	Nfkbiz
Gfer	Phb2
Pvr	Card9
Supt6	Ninj1
Casp4	N4bp1
Zfp683	Polr3c
Fut7	Tbk1
Cd1d1	Nmi
Ifng	Trim44
Cd226	Zdhhc1
Jak3	Ceacam1
Stxbp2	Gfi1
Il17a	Gbp2b
Stxbp1	Mefv
Cd5l	Dhx58
Kmt5c	Klrd1
Zfp35	Klrk1
Rc3h1	Trim5
Clec12b	Sarm1
Colec11	H2-T3
Arrb2	H2-T23
Adora2b	H2-T22
Serping1	H2-Q7
Serpinb9b	H2-Q6
Il20rb	H2-Q4
Cd300a	H2-Q2
Enpp3	H2-Q10
Ulbp1	H2-M3
Bcl6	H2-M2
Ptpn22	Traf3ip1
Ppp3cb	H2-K1
Il33	Cx3cl1
Lacc1	Usp27x
Malt1	Pqbp1
Zpbp2	Xrcc6
Smad7	Unc93b1
Epx	Sertad3
Klrb1a	Ddx3x
Klrb1b	Stat1
Klrb1c	Stat5a
Evpl	Stat5b
Socs5	Tarbp2
Rsad2	Ifi206
Ndfip1	Tlr11
Il18rap	Tlr12
Aplf	Tlr13
Lbp	Ptgs2os
Litaf	Trim62
Ptpn6	Polr3g
Cd36	Peli3
D6Wsu163e	Lrp8
Atad5	Nfkbil1
Fcer2a	Traf3ip2
Ap1g1	Syk

Tgfb2	Rnf216
Raet1e	Appl2
Bcr	Gfer
B2m	Pvr
Ccr2	Znrf1
Havcr2	Igtp
Atg9a	Myo1f
Irf1	Casp4
Spn	Oas1a
Itgb2l	Oas3
Arg1	Oasl1
Ccr7	Mmrn2
Apoa2	Gpr108
Atg5	Ptpn2
Raet1d	Ptprs
Nfkbid	Ifng
Klri1	Srebf1
Tirap	Cd226
Shld1	Creb3
Cxcl5	Ttll12
Dusp10	Il17a
Hfe	Epg5
Cd59a	Mapkapk3
Dpp4	Lrrfip2
Shld3	Bcl10
Appl1	Clec12b
Rc3h2	Chuk
Klrc2	Arrb2
Klrc1	Arf6
Lgals9	Optn
Zbtb7b	Ythdf3
Nod2	Ifih1
Mzb1	Serping1
Lilrb4a	Serpinb9b
Mr1	Sfpq
P2rx7	Cd300a
H60c	Ulbp1
Exosc3	Fosl1
Sphk2	Ptpn22
Nlrx1	Aurkb
H2-M5	Sin3a
H2-Q1	Lacc1
H2-T24	Lrrc14
Il4i1	Wdfy1
A2m	Clpb
Tlr2	Kcnj8
Stx7	Flot1
Cd59b	Nt5c2
Tbx21	Mul1
Clcf1	Irgm1
Fadd	Hcfc2
Vamp7	Klrb1a
Cd81	Zcchc3
Slamf1	Klrb1b
Pglyrp1	Klrb1c
Trp53bp1	Gata6
Klrb1f	Evpl
Mapkapk2	Ppp6c
Cd244a	Rsad2
Clec2d	Otud4
Shb	Ikbke
Spi1	Nagk
Unc13d	Dhx9
Ccl2	Il18rap
Ager	Itch
Stx4a	Lbp
Fcer1a	Cd36

Ms4a2		Adam8
Fcer1g		Ap1g1
Cfh		Akirin2
Cd40lg		Arg2
Cd40		Havcr2
Cd37		Cd300ld3
Ptafr		Mndal
Cd28		Irf2
Cd27		Irf1
Cd24a		Erbin
Cd22		Pja2
Slamf6		Eif2ak4
Foxp1		Src
Xbp1		Ipo5
Vav1		Spn
Tfrc		Treml4
Cuedc2		Arg1
Gab2		Il15
Cx3cr1		App
Cadm1		Apoe
Ccr6		Rftn1
Rps19		Atg5
Serpinb9		Irak1
Ddx60		Lyar
Vpreb3		Zdhhc5
Arid5a		Trafd1
Pagr1a		Akt1
Mill2		Trim12c
C3		Dnaja3
Btk		Ifnlr1
Tap2		Cactin
Tap1		Zdhhc18
Cd96		Klri1
Cr1l		Tirap
Crk		Tnip3
Tgfb3		Clec4n
Tgfb1		Ifi211
Masp1		Tifa
Cr2		Irf4
Lag3		Mark4
Hmces		Cxcl5
Map3k7		Dusp10
Gpi1		Ifi208
Cd84		Dpp4
Vamp8		Appl1
Fcgr1		Tigar
Fcgr2b		Klrc2
H60b		Klrc1
Hpx		Gbp2
Parp3		Lgals9
Nlrp3		Nod2
Plcg2		Prdx2
Prkcz		Ywhaz
Was		Trim25
Ahr		Cav1
Cfp		Mr1
Nr4a3		Trim12a
Tnfsf13		Eif2ak2
Bst2		Zbp1
Pnp		Znfx1
		Sash1
		Nlrx1
		Irak2
		Lrsam1
		Txk
		Tmem126a
		Samhd1

H2-M5
H2-Q1
Lrch4
Il4i1
A2m
Tlr2
Ube2k
Cd300lf
Ufd1
Fadd
Mettl3
Klrb1f
Mapkapk2
Clec2d
Stat2
Spi1
Ccl5
Plscr1
Colec12
Isg15
Nono
Cfh
Cd40lg
Cd40
Cd37
Cd24a
Cd14
Slamf6
Slc46a2
Foxp1
Vav1
Cnot7
Oas1c
Otulin
Rbm47
Ap3b1
Lsm14a
Polr3d
Alpk1
Cadm1
Rps19
Serpinb9
Rnf31
Ddx60
Nr1h3
Fpr2
Adar
Trim41
Peli1
Csnk1a1
Eif4e2
Btk
Tap2
Tap1
Bmp6
Tomm70a
Ilrun
Cd96
Crk
Polr3f
Tgfb1
Nlrc5
Lag3
Trim21
Riok3
Matr3
Cdc37

Map3k7
Mapk8
Mapk3
Sirt2
Nlrp1a
Cd84
Ifi35
Gbp5
Cyba
Trim30a
Hpx
Nlrp3
Plcg2
Pik3ap1
Ahr
Parp9
Apobec3
Cptp
Prkce
Prkca
Ifi213
Esr1
Dapk1
Dab2ip
Elmod2
Dhx33
Tspan32

T cells

GO_0002697_regulation.of.immune.effector.process	GO_0050863_regulation.of.T.cell.activation	GO_0002456_T.cell.mediated.immunity	GO_0001906_cell.killing	GO_0022407_regulation.of.cell.cell.adhesion	GO_0019221_cytokine.mediated.signaling.pathway	GO_0034341_response.to.type.II.interferon
Klk7	Ccl19	Foxp3	Ccl19	Map2k5	Xcr1	Ccl19
Ccl19	Dusp3	Slc11a1	Coro1a	Ccl19	Klf6	Cdc42ep2
Thoc1	Pde5a	Clec4g	Ccl8	Adamts18	Ccl19	Ifitm6
GO_0002697_re	Laptm5	Il7r	Camp	Ubash3b	Bbs2	Ccl8
Klrc3	Coro1a	Il12b	Nlrp6	Dusp3	Ifnz	Sirpa
Laptm5	Cd274	Il12a	Sh2d1b1	Pde5a	Il17rb	Il23r
Cxcl1	Sirpa	Il18	Fcgr4	Laptm5	Ebi3	Ciita
Dhx36	Tox	Icam1	Myd88	Specc1l	Ifitm6	Shfl
Il17f	Tnfsf11	Jag1	Cebpg	Coro1a	Cxcl1	Slc11a1
Shld2	Dhps	Cdh17	Sh2d1a	Cd274	Gab1	Slc22a21
Fzd5	Cd80	Ctsh	Atn1	Sirpa	Il17f	Tyk2
Traf2	Cd86	Cd46	Pomc	Tnfsf11	Ccl8	Rab11fip5
Msh2	Il4ra	H2-D1	Il7r	Dhps	Traf1	Gbp4
Cd177	Ripk2	Sash3	Ighg1	Serpine2	Traf2	Jak1
Cd80	Rara	Nfkbiz	Ncf1	Cd80	Fzd4	Evl
Cd86	Foxp3	Gzmb	Hmgn2	Cd86	Il23r	Ccl9
Angpt1	Rag1	H2-T23	Il18	Adam19	Tnfsf11	Actg1
C1qbp	Rac2	H2-K1	Lyz2	Il4ra	Il18r1	Syncrip
Sh2d1b1	Ptprc	Unc93b1	Il21	Il6ra	Il4ra	Stxbp4
Il18r1	Ripk3	Il31ra	Ltf	Rdx	Il6ra	Tnf
Il4ra	Pik3r6	Gzmm	Lgals3	Ripk2	Cxcr2	Ifitm2
Rbp4	Bid	Kdelr1	Rnf19b	Rara	Ripk2	Stx8
Ripk2	Fancd2	Lyst	Pglyrp4	Foxp3	Traf3	Casp1
Rara	Wnt10b	Stx11	Spag11b	Rag1	Myd88	Il12b
Rap1a	Lrrc32	Serpinb9b	Gbp7	Ptprc	Adipor2	Cd74
Foxp3	Scrib	Dlg1	Lyz1	Prkg1	Sirt1	Rpl13a
Rac2	Cd160	Kdm5d	Ccl28	Pik3r6	Adipor1	Tlr4
Nectin2	Tnfrsf13c	Ppp3cb	Trem1	Wnt10b	Ccr8	Daxx
Ptprc	Tyk2	Il18rap	Trem3	Lrrc32	Gpr35	Xcl1
Ripk3	Prkar1a	Aire	Gbp3	Scrib	Lepr	Cxcl16
Pik3r6	Rorc	Emp2	Irgm2	Cd160	Il1rap	Il12rb1
Ffar2	Hes1	Raet1e	Ctsh	Tnfrsf13c	Tnfrsf13c	Irf8
Pkn1	Cd55	Ebag9	Ccl22	Tyk2	Ptk2b	Jak2
Myd88	Cd55b	B2m	Cxcl13	Prkar1a	Tyk2	Cd47
Sirt1	Cd209b	Treml4	H2-D1	Hes1	Rabgef1	Gbp7
Cd160	Tnfrsf21	Rftn1	Fgl2	Cd55	Jak1	Vamp3
Rabgef1	Actb	Cd70	Fcgr3	Cd55b	Tnip2	Capg
Rif1	Pla2g2e	P2rx7	Tusc2	Mad2l2	Ccl9	Kif16b
Cd55	Abl2	Myo1g	Cxcl12	Jak1	Trem2	Gbp6
Cd55b	Abl1	Rab27a	Gzmb	Gtpbp4	Pias3	Actr2
Adora3	Smarcd1	Fosl2	Ninj1	Cd209b	Ackr2	Rab7b
Mad2l2	Smarcd2	Ctsc	Nkg7	Tnfrsf21	Il1rl2	Dapk3
Pms2	Il1rl2	Ephb6	Gbp2b	Actb	Tut4	Rps6kb1
Snx4	Zmiz1	Cd8a	Gapdh	Zdhhc2	Pdgfb	Gbp3
Ppl	Card11	Stard7	H2-T23	Pla2g2e	Oas1g	Ccl12
Ticam2	Drosha	Serpinb9	H2-K1	Abl2	Tnf	Ccl22
Panx1	Lat	Slfn2	Stat5a	Abl1	Osm	Myo18a
Mlh1	Smarcb1	Crlf2	Stat5b	Smarcd1	Nfkbia	H2-Aa
F2rl1	Blm	Tap2	Gzmm	Smarcd2	Ifitm2	Gsn
Acp5	Cd83	Cr1l	Prdx1	Il1rl2	Prlr	Acod1
Trem2	Cdkn2a	Cr2	Igtp	Zmiz1	Il9r	Actr3
Pld2	Adrm1	Prf1	Kif5b	Card11	Il7r	Ifitm7
Sh2d1a	Clec4g		Lyst	Klf4	Il7	Kynu
Slamf8	Prnp		Stxbp2	Gcnt2	Il6st	Gch1
Tnf	Il7r		Bcl2l11	Smarcb1	Il6	Gbp2b
Lta	Il7		Stx11	Blm	Il5ra	Gapdh
Prkdc	Il6st		Serpinb9b	Tnf	Il4	Mefv
Clec4g	Il6		Hamp	Cd83	Il3ra	H2-Q7
Susd4	Il4		Ulbp1	Ephb3	Il2rg	H2-Eb1

Pomc	Il2rg	Ppp3cb	Cdkn2a	Il2rb	Cx3cl1
Il7r	Il2ra	Emp2	Clec4g	Il2ra	H2-Ab1
Il6	Il2	Ptpn6	Prnp	Il2	Stat1
Il4	Il1b	Hc	Il7r	Il1r2	Ccl7
Il2	Il1a	Gzma	Il7	Il1r1	Igtp
Il1r1	Il12b	Gzmc	Il6st	Il1b	Kif5b
Casp1	Il12a	Raet1e	Il6	Il1a	Ifng
Il1b	Cd74	Ebag9	Il4	Il12b	Ifngr1
Il13	Ihh	B2m	Il2rg	Il12a	Ifngr2
Il12b	Kat5	Ccl17	Il2ra	Il10ra	Aqp4
Il12a	Traf6	Ccl25	Il2	Cd74	Stxbp2
Il10	Dusp22	Raet1d	Il1rn	Traf6	Stxbp1
Cd74	Igfbp2	Rpl30	Il1b	Tnfrsf11a	Stxbp3
Traf6	Igf2	Gbp2	Il1a	Tnfrsf1a	Stx11
Dusp22	Igf1	P2rx7	Il12b	Rraga	Rab12
Trpm4	Ido1	Rab27a	Il12a	Il18	Ifitm1
Ighm	Il27	H60c	Il10	Plvap	Mrc1
Ighg1	Rasgrp1	Cxcl14	Cd74	Lyn	Irgm1
Ighg2b	Hmgb1	Ctsc	Ihh	Il21r	Cdc42ep4
Igf2	Hlx	Vamp7	Kat5	Ikbkb	Ccl17
Il27	Il18	Bad	Traf6	Lifr	Slc22a5
Ncf1	Zc3h12a	Pglyrp1	Dusp22	Krt8	Ccl24
Clnk	Il23a	Unc13d	Taok2	Il15ra	Irf1
Rasgrp1	Il21	Ccl1	Igfbp2	Ccr1	Gbp10
Dnase1l3	Anxa1	Ccl27a	Igf2	Ccr3	Snca
Hmox1	Lgals3	F2	Igf1	Actn4	Ifitm3
Hmgb1	Lgals1	Cd2	Ido1	Ccr9	Ccl25
Hlx	Lef1	Romo1	Afdn	Xcl1	Dnaja3
Hk1	Lck	Rps19	Rasgrp1	Il12rb1	Slk
Klre1	Gimap3	Serpinb9	Hmgb1	Socs1	Gbp9
Il18	Xcl1	Arl8b	Hlx	Tnfrsf4	Vamp4
Zc3h12a	Il12rb1	C8g	Pik3r1	Tnfrsf1b	Rab20
Trim6	Socs1	C3	Il18	Lilra5	Gbp2
Il23a	Tnfsf4	Tap2	Zc3h12a	Nup85	Rab43
Lyn	Twsg1	Ctsg	Zdhhc21	Krt18	Zyx
Il21	Tnfrsf1b	Lag3	Mdga2	Kras	Pde12
Klrb1	Rasal3	Ppbp	Il23a	Kit	Sp100
Paxip1	Hsp90aa1	Fcgr1	Lyn	Jak2	Tlr2
Tlr4	Hspd1	H60b	Il21	Mpl	Ccl6
Anxa1	Hspb1	Gbp5	Anxa1	Hdac4	Ccl5
Tlr9	Jak2	Prf1	Fstl3	Ifnar2	Ccl4
Fbxo38	Cd47	Cxcl10	Lgals3	Aim2	Ccl3
Pdpk1	Itgal	Cxcl9	Lgals1	Cdip1	Ccl2
Lgals3	Mad1l1	Plekhm2	Spint2	Il12rb2	Ccl1
Slc15a4	Fanca		Lef1	Il16	Stx4a
Lamp1	Ripor2		Lck	Iigp1	Flnb
Gimap3	Sirpb1a		L1cam	Cxcr1	Cd40
Xcl1	Cd209e		Mdga1	Card14	Vim
Rapgef1	Cd209d		Gimap3	Ccl12	Trp53
Pglyrp4	Cd209c		Xcl1	Tnfsf18	Gbp8
Ptprj	Cd209a		Il12rb1	Fer	Camk2a
Pla2g3	Kitl		Socs1	Csf1r	Calm1
Tnfrsf4	Mdk		Tnfsf4	Csf3r	Cdc42
Tnfsf4	Ascl2		Twsg1	Csf2ra	Slc26a6
Tnfrsf1b	Pla2g2d		Fermt3	Csf1	Tgtp1
Rtn4	Zfp608		Emilin2	Csf2	Myo1c
Ticam1	Zbtb1		Icam1	Ccl22	Trim21
Hspd1	Tarm1		Zfp703	Cxcr3	Vamp8
Kmt5b	Cgas		Rasal3	Cxcl13	Gbp5
Kit	Gimap5		Hsp90aa1	Usp25	Vps26b
Cd47	Smarcc1		Hspd1	Pycard	Hpx
Itgb2	Arid1b		Hspb1	Irf5	Was
Itgam	Tnfsf18		Jak2	Irak3	Dapk1
Ankrd17	Nckap1l		Cd47	Rela	Bst2
Dnajb9	Cd46		Itgb2	Yap1	Nos2
Nod1	Selenok		Itgal	Fkbp1a	
Mif	Pycard		Itga6	Slc27a1	

Ascl2	Rps3	Itga4	Irf7
Scimp	H2-D1	Mad1l1	Fas
Mavs	H2-Aa	Ripor2	Irf3
Zbtb1	Btnl2	Sirpb1a	Oas1e
Cgas	Fgl2	Cd209e	Ackr3
Gimap5	Zbtb16	Cd209d	Cxcl12
Ube2j1	Tmem131l	Cd209c	Nfkbiz
Stap1	Lax1	Cd209a	Zc3h15
Tnfsf18	Fas	Kitl	Ackr1
Nckap1l	Zc3h12d	Mdk	Ifitm7
Ddx1	Erbb2	Mbp	Il22ra2
Pram1	Cyld	Ascl2	Ceacam1
Fer	Carmil2	Pla2g2d	Cx3cl1
Cd46	Lmo1	Ccl28	Foxc1
Spon2	Pten	Zfp608	Stat1
Gprc5b	Clec2i	Zbtb1	Stat4
Tyrobp	Crtam	Tarm1	Stat3
Myo18a	Cd6	Gimap5	Stat5a
Sema7a	Ildr2	Smarcc1	Stat5b
Pycard	Btla	Magi1	Stat6
Irf5	Dock8	Jag1	Flt3
H2-D1	Sash3	Ptpn23	Egr1
Grn	Nfkbiz	Arid1b	Sh2b2
Irak3	Gm5150	Tnfsf18	Il31ra
Prg2	Lgals8	Nckap1l	Il27ra
Fgl2	Tnfrsf14	Cd46	Cib1
Siglecg	Loxl3	Cxcl13	Csf2rb2
Fgr	Cblb	Selenok	Traf3ip2
Fes	Ceacam1	Pycard	Oasl2
Fcgr3	Ambra1	Rps3	Syk
Tlr7	Gata3	H2-D1	Ccl7
Tlr3	H2-T3	H2-Aa	Tnfsf13b
Pten	H2-T23	Gstp1	Appl2
Crtam	H2-T22	Btnl2	Oas1a
Ffar3	Vsir	Rela	Oas2
Klri2	H2-Q7	Fgl2	Oasl1
Tnfaip3	H2-Q6	Zbtb16	Ifnar1
Sash3	H2-Q4	Tmem131l	Ifng
Pglyrp2	H2-Q2	Lax1	Ifngr1
Nfkbiz	H2-Q10	Ptk2	Ifngr2
Phb2	H2-Ob	Ets1	Txndc17
Ddx21	H2-Oa	Zc3h12d	Il11
Card9	H2-DMb2	Erbb2	Il11ra1
Gm11127	H2-DMb1	Cyld	Jagn1
Tnfrsf14	H2-DMa	Carmil2	Rbm15
Loxl3	H2-M2	Pten	Jak3
Ceacam1	Fcho1	Crtam	Tjp2
Axl	H2-K1	Cd6	Il17ra
Dnase1	H2-Eb2	Tnfaip3	Il17a
Gata3	H2-Eb1	Cxcl12	Pirb
Gata2	Foxj1	Ildr2	Smad4
Gata1	H2-Ab1	Btla	Chuk
Dhx58	Stat5a	Dock8	Cebpa
Klrd1	Stat5b	Sash3	Numbl
Klrk1	Tespa1	Nfkbiz	Ifih1
H2-T3	Tnfsf14	Gm5150	Tnfrsf18
H2-T23	Icosl	Mex3b	Ext1
H2-T22	Prdm1	Lgals8	Bbs4
Vsir	Zap70	Mmrn1	Il20rb
H2-Q7	Smarca2	Tnfrsf14	Ifitm1
H2-Q6	Arid1a	Loxl3	Ccr5
H2-Q4	Syk	Cblb	Fosl1
H2-Q2	Tnfsf13b	Ceacam1	Sin3a
H2-Q10	Zeb1	Vegfa	Il33
H2-DMb2	Casp3	Ambra1	Mt3
H2-DMb1	Ap3d1	Gcnt1	Wbp1l
H2-M3	Smarcc2	Gata3	Traf5

H2-M2	Zfp683	H2-T3	Socs5
H2-K1	Ccdc88b	H2-T23	Ikbke
Foxj1	Ptpn2	H2-T22	Il18rap
Stat5a	Klhl25	Vsir	Ptpn6
Stat5b	Cd1d1	H2-Q7	Ccr4
Stat6	Ifng	H2-Q6	Tradd
Dennd1b	Jak3	H2-Q4	Il17re
6030468B19Rik	Runx1	H2-Q2	Ccr2
Il27ra	Cbfb	H2-Q10	Cntf
Tmbim6	Sirpb1b	H2-Ob	Ccl17
Ephb2	Zfp35	H2-Oa	Ccl24
Syk	Bcl10	H2-DMb2	Cnot9
Appl2	Dlg5	H2-DMb1	Irf1
Abr	Rc3h1	H2-DMa	Il17rc
Gfer	Rhoh	H2-M2	Src
Pvr	Cd276	Fcho1	Ccr7
Supt6	Adora2a	H2-K1	Il15
Casp4	Ankle1	H2-Eb2	Irak1
Zfp683	Il20rb	H2-Eb1	Ifitm3
Fut7	Dlg1	Cx3cl1	Mkks
Cd1d1	Cd300a	Foxj1	Tollip
Ifng	Bcl6	Nfat5	Ccl25
Cd226	Ptpn22	Pdpn	Akt1
Jak3	Ppp3ca	H2-Ab1	Cxcl3
Stxbp2	Malt1	Stat5a	Ifnlr1
Il17a	Glmn	Stat5b	Lims1
Stxbp1	Smad7	Epb41l5	Pira2
Cd5l	Dicer1	Fut4	Lilra6
Kmt5c	Irgm1	Tespa1	Tirap
Zfp35	Socs5	Mia3	Cxcl5
Rc3h1	Pag1	Mink1	Rps6ka5
Clec12b	Ndfip1	Tnfsf14	Gm13283
Colec11	Phf10	Icosl	Cd70
Arrb2	Itch	Zap70	Ilk
Adora2b	Ptpn6	Smarca2	Trim65
Serping1	Adam8	Arid1a	Appl1
Serpinb9b	Tsc2	Pde4d	Acsl1
Il20rb	Itpkb	Tnr	Ptprn
Cd300a	Zfp609	Syk	Cav1
Enpp3	Sit1	Tnfsf13b	Lilrb4a
Ulbp1	Nrarp	Casp3	Il10rb
Bcl6	Arg2	Ap3d1	Gfra2
Ptpn22	B2m	Smarcc2	Wnk1
Ppp3cb	Slc7a1	Myo10	Irak2
Il33	Ccr2	Ccdc88b	Cxcr5
Lacc1	Havcr2	Ptpn2	Cxcr4
Malt1	Irf1	Klhl25	Stk39
Zpbp2	Spta1	Fut7	Tnfrsf23
Smad7	Spn	Cd1d1	Rps6ka4
Epx	Sox4	Ifng	Cd300lf
Klrb1a	Sox13	Notch4	Ctr9
Klrb1b	Sox12	Jak3	Il20ra
Klrb1c	Sos2	F11r	Irak4
Evpl	Sos1	Magi2	Bad
Socs5	Kat2a	Runx1	Ccr10
Rsad2	Arg1	Cbfb	Epor
Ndfip1	Ccr7	Sirpb1b	Stat2
Il18rap	Il15	Zfp35	Srsf1
Aplf	Brd7	Bcl10	Spi1
Lbp	Dnaja3	Dlg5	Ccl6
Litaf	Adk	Ppm1f	Ccl5
Ptpn6	Ada	Rc3h1	Ccl4
Cd36	Tigit	Myadm	Ccl3
D6Wsu163e	Nfkbid	Rhoh	Ccl2
Atad5	Flot2	Cd276	Ccl1
Fcer2a	Dusp10	Adora2a	Sh2b3
Ap1g1	Icos	Il20rb	Dok1

Tgfb2	Efnb3	Dlg1	Tnfrsf17
Raet1e	Hfe	Cd300a	Fcer1g
Bcr	Cd59a	Bcl6	Trbv13-2
B2m	Gpam	Ccr5	F3
Ccr2	Dpp4	Ptpn22	Cd44
Havcr2	Rc3h2	Ppp3ca	Cd4
Atg9a	Tnfrsf9	B4galnt2	Oas1c
Irf1	Tnfsf9	Trpv4	St18
Spn	Lgals9	Malt1	Trp53
Itgb2l	Zbtb7b	Glmn	Ugcg
Arg1	Prdx2	Smad7	Cx3cr1
Ccr7	Cav1	Flot1	Ccr6
Apoa2	Efnb1	Irgm1	Csf2rb
Atg5	Lilrb4a	Kifap3	Crlf2
Raet1d	Tnfaip8l2	Socs5	Il1rl1
Nfkbid	Runx3	Piezo1	Csnk2b
Klri1	Slc4a1	Pag1	Il17rd
Tirap	Prelid1	Ndfip1	Commd7
Shld1	Pbrm1	Phf10	Cxcr6
Cxcl5	Dapl1	Itch	Pf4
Dusp10	Actl6a	Ptpn6	Ppbp
Hfe	Znhit1	Skap1	Map3k7
Cd59a	Nck1	Adam8	Mapk3
Dpp4	H2-M5	Tsc2	Ghr
Shld3	H2-Q1	Itpkb	Ccrl2
Appl1	Il4i1	Zfp609	Oxsr1
Rc3h2	Zc3h8	Nrarp	Prmt2
Klrc2	Pdcd1lg2	Arg2	Hpx
Klrc1	Mapk8ip1	B2m	Plcb1
Lgals9	Gli3	Slc7a1	Il13ra1
Zbtb7b	Cd59b	Ccr2	Cxcl10
Nod2	Tbx21	Havcr2	Cxcl9
Mzb1	Slc4a2	Irf1	Notch1
Lilrb4a	Fadd	Vps33b	Cxcl2
Mr1	Cd81	Src	
P2rx7	Nck2	Spta1	
H60c	Mettl3	Spn	
Exosc3	Slamf1	Sox4	
Sphk2	Clptm1	Sox13	
Nlrx1	Ephb6	Sox12	
H2-M5	Bad	Ass1	
H2-Q1	Cd244a	Arg1	
H2-T24	Sh3rf1	Ccr7	
Il4i1	Shb	Il15	
A2m	Ccl5	Irak1	
Tlr2	Ccl2	Efna5	
Stx7	Sh2b3	Ccl25	
Cd59b	Ager	Brd7	
Tbx21	Aif1	Alox5	
Clcf1	Sart1	Alox12	
Fadd	Cebpb	Alox15	
Vamp7	Cd5	Akt1	
Cd81	Cd44	Dnaja3	
Slamf1	Cd40lg	Adk	
Pglyrp1	Cd4	Ada	
Trp53bp1	Cd3e	Tigit	
Klrb1f	Cd37	Nfkbid	
Mapkapk2	Cd28	Mapk7	
Cd244a	Cd27	Bmp7	
Clec2d	Cd24a	Flot2	
Shb	Slc46a2	Dusp10	
Spi1	Tcf7	Icos	
Unc13d	Egr3	Efnb3	
Ccl2	Xbp1	Hfe	
Ager	Arid2	Cd59a	
Stx4a	Scgb1a1	Gpam	
Fcer1a	Tfrc	Dpp4	

Ms4a2	Ap3b1	Rc3h2
Fcer1g	Nkap	Tnfsf9
Cfh	Pawr	Lgals9
Cd40lg	Gpnmb	Zbtb7b
Cd40	Ccr6	Prdx2
Cd37	Smarcd3	Plpp3
Ptafr	Slfn1	Cav1
Cd28	Vcam1	Efnb1
Cd27	Sirpb1c	Lilrb4a
Cd24a	Socs6	Tnfaip8l2
Cd22	Peli1	Runx3
Slamf6	Ctnnb1	Slc4a1
Foxp1	Smarca4	Pbrm1
Xbp1	Braf	Dapl1
Vav1	Bmi1	Wnk1
Tfrc	Ctsg	Actl6a
Cuedc2	Ctla4	Nck1
Gab2	Ctla2a	H2-M5
Cx3cr1	Csk	H2-Q1
Cadm1	Iglc4	Il4i1
Ccr6	Thy1	Mapk14
Rps19	Tgfbr2	Lrfn3
Serpinb9	Tgfb1	Zc3h8
Ddx60	Lag3	Pdcd1lg2
Vpreb3	Cyp26b1	Celsr2
Arid5a	Smarce1	Gli3
Pagr1a	Rhoa	Cd59b
Mill2	Gnrh1	Tbx21
C3	Dtx1	Slc4a2
Btk	Nlrp3	Rap1gap
Tap2	Prkcz	Fadd
Tap1	Prkcq	Cd81
Cd96	Sdc4	Nck2
Cr1l	Efnb2	Slamf1
Crk	Hsph1	Ephb6
Tgfb3	Pnp	Bad
Tgfb1	Tspan32	Dennd6a
Masp1		Cd244a
Cr2		Ank3
Lag3		Shb
Hmces		Spi1
Map3k7		Selp
Gpi1		St3gal4
Cd84		Sele
Vamp8		Ccl5
Fcgr1		Ccl2
Fcgr2b		Sh2b3
H60b		Ager
Hpx		Aif1
Parp3		Sart1
Nlrp3		Cebpb
Plcg2		Cdh1
Prkcz		Cd9
Was		Cd5
Ahr		Cd44
Cfp		Cd40lg
Nr4a3		Cd4
Tnfsf13		Cd3e
Bst2		Cd37
Pnp		Ptafr
		Cd28
		Cd27
		Cd24a
		Cited2
		Egr3
		Xbp1
		Wnt4

Arid2
Scgb1a1
Tfrc
Ap3b1
Nkap
Adtrp
Pawr
Adipoq
Gpnmb
Smarcd3
Gp1ba
Slfn1
Vcam1
Sirpb1c
Socs6
Peli1
Akna
Capn1
Mfsd2b
Smarca4
Bmp6
Bmp2
Bmi1
Epha7
Ctsg
Ctla4
Iglc4
Tjp1
Thy1
Tgfbr2
Tgfb1
Lag3
Epcam
Swap70
Map2k1
Smarce1
Rhoa
Gnrh1
Rgcc
Dtx1
Nlrp3
Plaur
Prkcz
Prkcq
Sdc4
Efnb2
Hsph1
Prkcd
Prkca
Chst2
Nr4a3
Pnp
Notch1
Nf2
Tspan32

**negative regulation of immune response**	**Negative regulation of T cell activation**	**Negative regulation of cell activation**
Nlrp4f	Adora2a	Adamts18
Banf1	Anxa1	Adamts18
Spink5	Anxa1	Ubash3b
Thoc1	Arg1	Thoc1
H2-M9	Arg1	Dusp3
H2-M9	Arg2	Dusp3
H2-M10.4	Ascl2	Pde5a
H2-M10.4	Ascl2	Laptm5
H2-M10.5	Ascl2	Laptm5
H2-M10.5	Bcl6	Laptm5
H2-M10.1	Bmp4	Rian
H2-M10.1	Bmp4	Rian
Parp14	Btla	Rian
Parp14	Btla	Cd274
H2-M11	Btn1a1	Cd274
H2-M11	Btn2a2	Cd274
Usp15	Btn2a2	Cd274
Foxf1	Casp3	Cd274
Cd80	Cbfb	Sfrp1
Atg12	Cblb	Sfrp1
Smpdl3b	Cd24a	Pla2g5
Nlrp6	Cd37	Rassf5
Nlrp6	Cd44	Foxf1
Trim27	Cd44	Serpine2
Sh2d1b1	Cd74	Serpine2
Il4ra	Cd80	Cd80
Foxp3	Cd86	Cd86
Foxp3	Cd274	Il4ra
Foxp3	Cd274	Foxp3
Foxp3	Cd274	Foxp3
Ptprc	Cd274	Foxp3
Npy5r	Cd274	Foxp3
Cd160	Cd276	Foxp3
Zp3r	Cd276	Foxp3
Zp3r	Cd300a	Foxp3
Serpinb9g	Cdkn2a	Foxp3
Rabgef1	Ceacam1	Foxp3
Gpx1	Ceacam1	Rag2
Gpx1	Ceacam1	Prkg1
Pdcd1	Cebpb	Pkn1
Pdcd1	Clec4g	Lrrc32
Cd55	Clec4g	Scrib
Cd55	Clec4g	Gclc
Cd55b	Crtam	C1qtnf1
Cd55b	Ctla4	C1qtnf1
Tyro3	Ctla4	Rabgef1
Mmp12	Ctla4	Rabgef1
H2-M10.2	Ctsg	Prkar1a
H2-M10.2	Cyld	Tyro3
H2-M10.6	Dapl1	Tyro3
H2-M10.6	Dlg1	Tnfrsf13b
Gigyf2	Dlg5	Tnfrsf13b
Trem2	Dtx1	Tnfrsf21
Wdr41	Dusp3	Tnfrsf21
Oas1b	Dusp3	Tnfrsf21
Oas1g	Dusp22	Tnfrsf21
Slamf8	Erbb2	Pla2g2f
Trex1	Fgl1	Pla2g2f
Trex1	Fgl1	Pla2g2e

Prkdc	Fgl2	Trem2
Clec4g	Foxj1	Trem2
Clec4g	Foxj1	Pdgfra
Clec4g	Foxp3	Pdgfb
Susd4	Foxp3	Pdgfa
Susd4	Foxp3	Cdkn2a
Susd4	Foxp3	Cdkn2a
Susd4	Foxp3	Clec4g
Pparg	Foxp3	Clec4g
Il7r	Gimap3	Clec4g
Il4	Gimap5	Prnp
Il4	Gli3	Pparg
Il2	Glmn	Il4
Il12b	Gnrh1	Il4
Il10	Gpnmb	Il4
Nlrp4e	Gpnmb	Il4
Dusp22	H2-Aa	Il4
Igf2	Havcr2	Il2ra
Nlrp4b	Havcr2	Il2ra
Ythdf2	Hfe	Il2
Nlrc3	Hlx	Il2
Hmox1	Hlx	Il10
Hlx	Hmgb1	Il10
Hlx	Hspb1	Cd74
Klre1	Ido1	Ihh
Zc3h12a	Ido1	Ihh
Smcr8	Ifnb1	Ihh
Anxa1	Ihh	Dusp22
Anxa1	Ihh	Ido1
Lgals3	Ihh	Ido1
Usp38	Il2	Clnk
Ccr1	Il2ra	Clnk
Pglyrp4	Il4	Nr1d1
Tnfsf4	Il4	Nr1d1
Vsig4	Il4	Hmox1
Vsig4	Il4i1	Hmgb1
H2-Bl	Il4i1	Hlx
H2-Bl	Il4i1b	Hlx
H2-Bl	Il4i1b	Zc3h12a
Npy	Il4ra	Lyn
Serpinb9h	Il20rb	Lyn
Dtx4	Ildr2	Anxa1
Psmb4	Ildr2	Anxa1
Nlrp4c	Irf1	Rhbdd3
Ascl2	Irf1	Lgals3
Ascl2	Itch	Lgals3
Ascl2	Jak3	Ldlr
Cgas	Jak3	Ldlr
Pcbp2	Lag3	Tbc1d10c
Tnfsf18	Lag3	Tbc1d10c
Nckap1l	Lag3	Cnr2
Fam3a	Lag3	Pla2g2a
Fer	Laptm5	Gimap3
Cd46	Laptm5	Cnr1
Cep63	Lax1	Pglyrp4
Olfm4	Lax1	Socs1
H2-D1	Lgals3	Tnfsf4
H2-D1	Lgals9	Tnfsf4
Grn	Lgals9	Twsg1
Grn	Lilrb4a	Twsg1
Irak3	Lilrb4a	Id2
Nlrp4a	Lilrb4a	Id2
Fgl2	Lilrb4b	Id2
Acod1	Lilrb4b	Vsig4
Psma1	Lilrb4b	Vsig4
Psma1	Loxl3	Hspb1
Samsn1	Lrrc32	Tafa3

Oas1e	Mad1l1	Tafa3
Gpr17	Marchf7	Mad1l1
Gpr17	Mdk	mCEACAM1a/iso:4L/SigPep-/NGlycoAsn+
Tnfaip3	Nckap1l	mCEACAM1a/iso:4L/SigPep-/NGlycoAsn+
Pglyrp2	Ndfip1	Ripor2
Parp1	Nfkbid	Ripor2
Parp1	Nrarp	Mertk
Pglyrp3	Pag1	Mertk
Tnfrsf14	Pag1	Mdk
Nmi	Pawr	Ascl2
Nmi	Pdcd1lg2	Ascl2
Loxl3	Pdcd1lg2	Ascl2
Ceacam1	Pdcd1lg2	Pla2g2d
Dhx58	Pdcd1lg2	Pla2g2d
Dhx58	Pde5a	Pla2g2d
Klrd1	Peli1	Zfp608
Klrd1	Peli1	Tarm1
Selenos	Pla2g2a	Tarm1
H2-T3	Pla2g2d	Gimap5
H2-T3	Pla2g2d	Emilin1
H2-T23	Pla2g2d	Emilin1
H2-T23	Pla2g2e	Lrfn5
H2-T22	Pla2g2f	Tnfsf18
H2-T22	Pla2g2f	Nckap1l
H2-T10	Pla2g5	Fer
H2-T10	Prdx2	Tyrobp
Vsir	Prkar1a	Tyrobp
H2-Q7	Prnp	Tyrobp
H2-Q7	Pten	H2-Aa
H2-Q6	Ptpn2	Grn
H2-Q6	Ptpn6	Grn
H2-Q4	Ptpn6	Syt11
H2-Q4	Ptpn22	Fgl2
H2-Q2	Ptpn22	Fgr
H2-Q2	Ptpn22	Tmem131l
H2-Q10	Rag2	Lax1
H2-Q10	Rc3h1	Lax1
H2-M2	Rc3h1	Samsn1
H2-M2	Rc3h1	Samsn1
H2-M1	Rc3h2	Marchf7
H2-M1	Ripor2	Fas
H2-L	Ripor2	Bpi
H2-L	Runx1	Bpi
H2-K1	Runx3	Zc3h12d
H2-K1	Scgb1a1	mSkint2
Foxj1	Scrib	mSkint2
Foxj1	Sdc4	Erbb2
Stat6	Sftpd	Pten
Il27ra	Shh	Pten
Il27ra	Shh	Crtam
Abr	Shh	Tnfaip3
Gfer	Skint2	Ildr2
Oas1a	Skint2	Ildr2
Oas1f	Slc4a2	Btla
Oas1h	Slc4a2	Btla
Oas3	Slfn1	Btla
Ptpn2	Smad7	Pglyrp2
Ptpn2	Socs1	Pglyrp3
Ptpn2	Socs5	Tnfrsf14
Ptpn2	Socs6	Tnfrsf14
Ifnb1	Spn	Loxl3
Jak3	Spn	Cblb
Ttll12	Spn	Ceacam1
Inpp5d	Spn	Ceacam1
Zfp35	Spn	Axl
Zfp35	Tarm1	Axl
Svep1	Tarm1	Btn1a1

Rc3h1	Tbx21	Pibf1
Rc3h1	Tbx21	Gal
Rc3h1	Tgfb1	Vsir
Clec12b	Tgfb1	Vsir
Arrb2	Tigit	Vsir
Ythdf3	Tigit	Vsir
Serping1	Tigit	Cx3cl1
Serpinb9e	Tigit	Foxj1
Serpinb9f	Tmem131l	Foxj1
Serpinb9c	Tnfaip8l2	Foxj1
Serpinb9b	Tnfaip8l2	Fn1
Serpinb9d	Tnfrsf14	Fn1
H2-M10.3	Tnfrsf14	Flt3
H2-M10.3	Tnfrsf21	Abr
Muc4	Tnfrsf21	Casp3
Il20rb	Tnfsf4	Casp3
Cd300a	Tnfsf4	Hmgb3
Cd300a	Tnfsf18	Ptpn2
Cd300a	Tsc2	Ifnb1
Enpp3	Tspan32	Vtcn1
Dcst1	Twsg1	Vtcn1
Bcl6	Twsg1	Vtcn1
Bcl6	Vsig4	Jak3
Bcl6	Vsig4	Jak3
Bcl6	Vsir	Inpp5d
Bcl6	Vsir	Inpp5d
Aurkb	Vsir	Runx1
Ppp3cb	Vsir	Cbfb
Il33	Vtcn1	Zfp35
Smad7	Vtcn1	Cd200
Smad7	Vtcn1	Dlg5
Smad7	Zbtb7b	Rc3h1
Mul1	Zbtb7b	Rc3h1
Mul1	Zbtb7b	Rc3h1
Klrb1b	Zbtb7b	Rc3h1
Klrb1b	Zc3h8	Cd276
Klrb1b	Zc3h12a	Cd276
Klrb1b	Zc3h12d	Adora2a
Klrb1b	Zfp35	Il20rb
Ppp6c	Zfp608	Dlg1
Socs5		Atm
Ndfip1		Cd300a
Ndfip1		Cd300a
Ptpn6		Cd300a
Ptpn6		Cd300a
Arg2		Cd300a
Otop1		Cd300a
Bcr		Cd300a
Col3a1		Enpp3
Ccr2		Bcl6
Ccr2		Bcl6
Havcr2		Bcl6
Havcr2		Bcl6
Havcr2		Ptpn22
Havcr2		Ptpn22
Havcr2		Ptpn22
Irf1		Glmn
Ins2		mCEACAM1a/iso:4L
Ins1		Smad7
Spn		Socs5
Arg1		Socs5
Arg1		Pag1
Atg5		Pag1
Fcrlb		Ndfip1
Fcrlb		Ndfip1
Lyar		Zbtb46
Trafd1		Itch

Trafd1	mCYLD/iso:4
Alox15	Ptpn6
Akt1	Ptpn6
Dnaja3	Ptpn6
Cactin	Btn2a2
Cactin	Btn2a2
Zdhhc18	Milr1
Dusp10	Tsc2
Hfe	Tsc2
Cd59a	Nrarp
Cd59a	Arg2
Cd59a	Bcr
Cd59a	Ccr2
Cd59a	Havcr2
H2-T-ps	Havcr2
H2-T-ps	Havcr2
Drd2	Havcr2
Rc3h2	Havcr2
Lgals9	Irf1
Zbtb7b	Irf1
Nod2	Spn
Nod2	Spn
Ywhaz	Spn
Lilrb4b	Spn
Lilrb4a	Spn
Lilrb4a	Sox11
Lilrb4a	Arg1
Lilrb4a	Arg1
Lilrb4a	Apoe
Lilrb4a	Alox12
Nlrx1	Tigit
Samhd1	Tigit
Samhd1	Tigit
H2-M5	Tigit
H2-M5	Nfkbid
H2-Q1	mPRDM1/iso:2
H2-Q1	mPRDM1/iso:1
Il4i1	Adgrf5
Il4i1	Hfe
Il4i1	Rc3h2
Il4i1b	Fgl1
A2m	Fgl1
Cd59b	Lgals9
Cd59b	Lgals9
Cd59b	Lgals9
Cd59b	Lgals9
Cd59b	Zbtb7b
Tbx21	Zbtb7b
Tbx21	Zbtb7b
Tbx21	Zbtb7b
Tbx21	Prdx2
C9orf72	Lilrb4b
Mettl3	Lilrb4b
Slamf1	Lilrb4b
Pglyrp1	Lilrb4b
Clec2d	Lilrb4a
Clec2d	Lilrb4a
Clec2d	Lilrb4a
Stat2	Lilrb4a
Stat2	Lilrb4a
Spi1	Lilrb4a
Isg15	Lilrb4a
Sh2d1b2	Lilrb4a
Oas1d	Lilrb4a
Cnot7	Lilrb4a
Oas1c	Lilrb4a
Il13ra2	Lilrb4a

Mkrn2	Tnfaip8l2
Rps19	Tnfaip8l2
Gpx2	Fbxo7
Gpx2	Fbxo7
Serpinb9	Runx3
Adar	Bank1
Eif4e2	Bank1
C4bp	Dapl1
C4bp	Il4i1
Tap2	Il4i1
Tap1	Il4i1b
Il1rl1	Il4i1b
Cd96	Zc3h8
Cr1l	Pdcd1lg2
Cr1l	Pdcd1lg2
Cr1l	Pdcd1lg2
Crk	Pdcd1lg2
Tgfb1	Gli3
Nlrc5	Tbx21
Nlrc5	Tbx21
Nlrc5	Cd300lf
Masp1	Cd300lf
Cr2	Slc4a2
Trim21	Slc4a2
Cd84	Gp5
Fcgr2b	Gp5
Fcgr2b	Pglyrp1
Fcgr2b	Lst1
Fcgr2b	Shh
Fcgr2b	Shh
Fcgr2b	Shh
Fcgr2b	Spi1
Parp3	Sh2b3
Adcyap1	Cebpb
Ahr	Cd9
	Tff2
	Cd44
	Cd44
	Cd37
	Cd37
	Cd24a
	Cd24a
	Scgb1a1
	Cst7
	Cst7
	Il13ra2
	Pawr
	Pawr
	Gpnmb
	Gpnmb
	Slfn1
	Nr1h3
	Socs6
	Peli1
	Peli1
	Btk
	Btk
	Bmp4
	Bmp4
	Gper1
	Ctsg
	Ctla4
	Ctla4
	Ctla4
	Ctla4
	Tgfb1
	Tgfb1

Cygb
Lag3
Lag3
Lag3
Lag3
Sftpd
Gnrh1
Cd84
Cd84
Fcgr2b
Fcgr2b
Dtx1
Parp3
Sdc4
Prkcd
Tspan32
Tspan32
